# SurroDock: A Deep Learning Surrogate for Accelerated Pre-Docking Ligand Prioritization in Structure-Based Virtual Screening

**DOI:** 10.3390/ijms27156663

**Published:** 2026-07-26

**Authors:** Jongkeun Choi

**Affiliations:** Department of Chemical & Biological Engineering, Chungwoon University, Incheon 22100, Republic of Korea; jkchoi@chungwoon.ac.kr

**Keywords:** surrogate modeling, deep learning, virtual screening, docking acceleration, estrogen receptor alpha, AutoDock Vina, molecular docking, applicability domain, early enrichment

## Abstract

The rapid expansion of make-on-demand and public chemical libraries has made exhaustive docking-based structure-based virtual screening increasingly difficult. This study introduces SurroDock, a lightweight deep-learning surrogate designed to approximate AutoDock Vina docking scores from low-cost two-dimensional molecular features, serving as a practical pre-filter for docking. SurroDock was evaluated for estrogen receptor alpha using two distinct conformations: an agonist-bound (PDB ID: 1GWR) and an antagonist/SERM-bound (PDB ID: 3ERT). The dataset comprised approximately 334,000 unique compounds curated from the NCI Open Database, PubChem, and BindingDB, all docked using a standardized AutoDock Vina workflow. The model was trained on concatenated 2D molecular representations comprising Morgan fingerprints, MACCS keys, RDKit physicochemical descriptors, Vina-inspired ligand descriptors, atom-pair fingerprints, and 2D pharmacophore fingerprints. The docking-score distributions differed substantially between receptor states, with 3ERT exhibiting more favorable scores than 1GWR and weak inter-state score correlation supporting state-specific modeling. Using the integrated Unified-200k training set (200,000 compounds randomly sampled per receptor from the three docked sources), SurroDock achieved strong held-out validation performance, with *R*^2^ values of approximately 0.88 for 1GWR and 0.93 for 3ERT. In retrospective screening-style evaluation, SurroDock recovered substantial fractions of Vina’s top-ranked compounds at the top-1% recall (Recall@1%) of approximately 0.57 and 0.61 for 1GWR and 3ERT, respectively, yielding corresponding enrichment factors (EF@1%) of approximately 57-fold and 61-fold relative to random selection. Overall, the results indicate that 2D-based docking-score surrogate modeling can provide a reproducible and retrainable strategy for large-scale structure-based virtual screening by concentrating docking resources on a smaller, enriched subset of compounds. Because SurroDock emulates a docking scoring function rather than experimental binding affinity, its predictions should be used as prioritization aids and complemented by confirmatory docking, pose inspection, and experimental validation.

## 1. Introduction

The synthetically accessible chemical space for small-molecule drug discovery has expanded to tens to hundreds of billions of candidate molecules, increasing the strategic value of computation-driven screening methods that can rapidly and selectively explore this vast landscape [1,2,3]. This expansion is mirrored in the rapid growth of public repositories, which now contain over 100 million unique structures, along with extensive bioactivity records. As of February 2026, PubChem indexes approximately 123 million unique compounds, 299 million bioactivity records, 44 million literature citations, and 83 million patents, underscoring the continued expansion of the publicly accessible chemical corpus [4]. Complementing these resources, public purchasing-oriented infrastructures such as ZINC-22 support access to ultra-large, readily procurable libraries [5], while industrial make-on-demand catalogs such as Enamine’s REAL Database provide drug-like chemical spaces at scales exceeding 10 billion compounds [6]. These entries are annotated with procurement and synthesis-effort metadata, enabling rapid hypothesis testing and analog retrieval.

Within this landscape, virtual screening operates as a cost-efficient prioritization strategy. Compared with high-throughput screening, which requires substantial infrastructure, consumables, and operational resources [7], virtual screening enables computational down-selection based on compound ranking. Rather than supplanting iterative wet-lab campaigns, virtual screening serves as a computational pre-filter that reduces the number of compounds requiring experimental confirmation to a physically manageable scale. Structure-based virtual screening (SBVS) has consequently become a central tool in early-stage drug discovery, leveraging three-dimensional target protein structures together with molecular docking and empirical or physics-inspired scoring functions to prioritize candidate ligands for experimental evaluation [8,9,10]. Docking-based prioritization can also be followed by downstream computational assessment, such as pose inspection, interaction analysis, and optional simulation-based refinement, to evaluate candidate plausibility and support further prioritization in virtual-screening workflows [11]. However, although docking methods are computationally efficient relative to more rigorous physical simulations, their scoring functions remain approximate, imperfect, and sometimes biased.

Make-on-demand libraries and open platforms have demonstrated that billion-scale virtual screens are technically feasible. Studies involving docking campaigns at the 10^8^–10^9^ scale have reported prospective identification of new chemotypes and potent active compounds [12,13]. Despite these successes, such workflows remain computationally and operationally demanding. For example, VirtualFlow screened approximately 1 billion compounds in roughly two weeks using approximately 10,000 CPU cores [12]. Although AutoDock Vina and subsequent refinements have improved search algorithms, scoring consistency, and usability [8,9], ultra-large screening campaigns, particularly those involving hundreds of thousands to tens of millions of molecules across multiple receptor conformations or functional states, still incur substantial cumulative costs. These bottlenecks intensify in scenarios involving cross-docking for selectivity profiling, multi-conformational panels to reflect structural flexibility, and rapid iterative ideation cycles that demand short design–evaluation turnaround times [2,3].

In this context, machine learning models based on low-cost molecular representations offer a practical approach to accelerating large-scale compound prioritization. While models trained directly on experimental affinity labels are desirable in principle, assembling large, target-consistent datasets is often limited by assay heterogeneity, inconsistent target conditions, and sparse availability of target-specific labels [14,15]. Moreover, public activity annotations including Ki and IC_50_ can exhibit substantial experimental uncertainty and inter-laboratory variability, further complicating supervised learning [15]. A practical alternative is to train machine learning models to approximate docking scores directly from inexpensive molecular representations. Docking provides protocol-consistent labels at scale whenever a target structure and docking workflow are available, enabling the stable compilation of target-specific training corpora at the 10^5^–10^6^ scale, despite the known limitations of docking scores as approximations to binding affinity [8,9,10,16,17]. This approach is related to the broader development of machine learning-based protein–ligand scoring functions, from RF-Score to more recent learning-based scoring methods [16,17]. However, in contrast to general scoring functions, docking-score surrogates are designed to emulate the output of a specific docking protocol, thereby enabling rapid, retrainable screening within a defined computational workflow.

Surrogate modeling of docking scores offers several practical advantages. Even though docking scores are heuristic, physics-informed proxies, accurately approximating them can enable pre-filtering of large libraries, reserving full docking for a smaller, enriched subset of compounds. This can reduce computational cost and provide rapid feedback during iterative exploration of large or less-explored chemical subspaces. However, building a reliable docking-score surrogate differs from traditional affinity-based quantitative structure–activity relationships. Docking outputs can vary across receptor conformations, inherit systematic biases from the scoring function, and include rare extreme values arising from pose generation, protonation or tautomeric states, ligand preparation, or receptor preparation artifacts. Addressing these sources of variance and bias is essential for robust down-selection and reproducibility. Deep Docking [18] has demonstrated that models trained on an initially docked subset can approximate docking outcomes for non-docked molecules, enabling substantial library contraction while retaining top-ranked candidates through an iterative active-learning loop that alternates between docking and classification-based re-ranking of hit/non-hit probability. This strategy exemplifies a broader class of machine learning-assisted virtual screening approaches that couple limited docking with fast model inference to achieve scalable prioritization.

Motivated by prior work on deep learning-based estrogen receptor (ER) activity prediction [19], this study extends the use of ligand-based molecular representations from binary activity classification to docking-score surrogate regression for SBVS. This study introduces SurroDock v1.0, a reproducible framework that approximates AutoDock Vina scores using two-dimensional molecular features, thereby providing a low-cost pre-docking triage mechanism for large chemical libraries. Unlike Deep Docking’s iterative classification loop, SurroDock directly regresses the continuous docking score in a single training pass on a fixed, pre-docked corpus, enabling fine-grained, conformation-specific ranking without repeated re-docking cycles. SurroDock was evaluated using ERα as a benchmark target across two functionally distinct receptor conformations: an agonist-bound state and an antagonist/selective estrogen receptor modulators (SERMs)-bound state. The framework emphasizes scaffold-aware validation, held-out evaluation, early enrichment analysis, and applicability-domain diagnostics to assess its robustness for downselection. By combining scaffold-aware validation with applicability-domain-guided interpretation, SurroDock aims to improve the reliability and diversity of top-ranked candidate sets. Overall, this study evaluates whether docking-score surrogate modeling can reduce full docking workloads while retaining high-ranking candidates for subsequent confirmatory docking, pose inspection, and experimental assays.

## 2. Results and Discussion

### 2.1. Docking Results from AutoDock Vina and Early Hit Identification

AutoDock Vina was used to generate reference docking-score datasets for the agonist-bound and SERM/antagonist-bound conformations of ERα. Before generating the large-scale training dataset for the surrogate model (SurroDock), the docking protocol was validated by redocking the co-crystallized ligands into their corresponding receptor structures: 1GWR (agonist) and 3ERT (SERM/antagonist).

The validation simulations successfully reproduced the favorable docking scores of the native ligands, yielding −10.7 kcal/mol for Estradiol (1GWR) and −9.8 kcal/mol for 4-hydroxytamoxifen (3ERT). In terms of structural accuracy, Estradiol exhibited an exceptional match to the crystal structure, with a root mean square deviation (RMSD) of 0.721 Å (Figure S1). 4-Hydroxytamoxifen showed a slightly higher RMSD of 1.250 Å; however, this deviation is primarily attributed to the conformational flexibility of its side chain rather than docking inaccuracy. Unlike the rigid steroid core of estradiol (two active torsions), 4-hydroxytamoxifen possesses a flexible tail (nine active torsions) that extends into the solvent-exposed region. This lack of steric constraint allows for greater motional freedom in the tail, accounting for the observed structural deviation. Collectively, the favorable docking scores and low RMSD values support the suitability of the docking protocol for generating internally consistent docking-score datasets.

**Figure 1:**
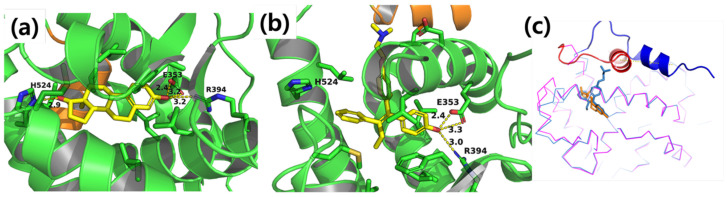
Structural states of the ERα ligand-binding domain (LBD). (**a**) Agonist-bound complex (PDB ID: 1GWR, with 17β-estradiol; ligand carbon atoms shown in yellow) showing Helix 12 (orange) in the closed active position, where it caps the ligand-binding pocket and forms the coactivator-binding activation function-2 (AF-2) surface. Conserved polar anchors, including Glu353, Arg394, and His524, together with the surrounding hydrophobic shell, stabilize the active conformation. (**b**) Antagonist-bound complex (PDB ID: 3ERT, with 4-hydroxytamoxifen; ligand carbon atoms shown in yellow), in which the bulky side chain displaces Helix 12 (orange) into an alternative antagonist orientation that occludes the AF-2 coactivator cleft while maintaining the conserved phenolic hydrogen-bonding network. (**c**) Superposition of the agonist-bound structure 1GWR (pink, with Helix 12 in red) and the antagonist-bound structure 3ERT (cyan, with Helix 12 in blue), highlighting Helix 12 relocation and accompanying pocket reshaping that underlie the switch between coactivator-competent and inhibitory receptor architectures.

After protocol validation, the prepared compound library was docked to the ERα ligand-binding domain (LBD) in both conformational states. The distinction between these states is critical, as the ERα LBD exhibits ligand state–dependent repositioning of Helix 12 (H12), which remodels the effective pocket volume and the activation function-2 surface (Figure 1). In the agonist-bound 1GWR, H12 adopts a closed active conformation that supports coactivator engagement and preserves the phenolic anchoring network (phenolic OH–Glu353/Arg394 and secondary OH–His524). In contrast, in the SERM-bound 3ERT, the bulky scaffold and side chain displace H12 into an alternative pose that occludes activation function-2 and expands hydrophobic occupancy within the cavity.

**Figure 2:**
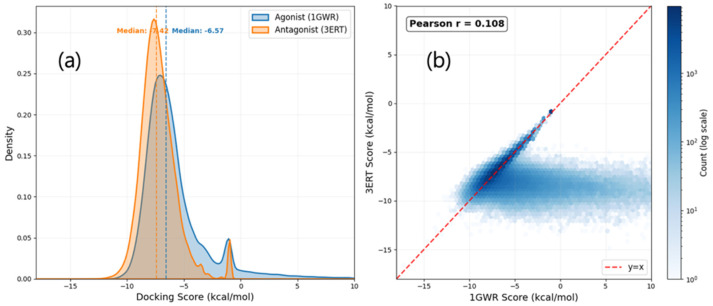
Large-scale docking comparison of ERα agonist-bound 1GWR and antagonist-bound 3ERT conformations. (**a**) KDE distributions of AutoDock Vina docking scores show a left shift for 3ERT relative to 1GWR, with median scores of −7.42 and −6.57 kcal/mol, respectively, consistent with enhanced ligand accommodation in the Helix 12-displaced antagonist state. (**b**) Hexbin correlation plot of compounds docked against both conformations. The red dashed line denotes the identity line (*y* = *x*). The low Pearson correlation (r =0.108) and density below the diagonal indicate weak score concordance and generally more favorable docking in 3ERT.

Figure 2 summarizes the comparative analysis of the docking results. The Vina docking-score distribution for the antagonist conformation (3ERT) is notably left-shifted relative to the agonist conformation (1GWR), with median values of −7.42 and −6.57 kcal/mol, respectively (Figure 2a). This quantitative shift (−0.85 kcal/mol difference in median) is consistent with the structural expansion of the hydrophobic pocket in the antagonist state, thereby allowing enhanced nonpolar contacts.

Furthermore, a pairwise comparison of docking scores for the intersecting ligand library reveals a remarkably low linear correlation (Pearson *r* = 0.108, Figure 2b). As illustrated in the scatter plot, most data points fall below the identity line (*y* = *x*), confirming that most ligands achieve more favorable binding energies in the antagonist conformation. The lack of a strong correlation indicates that the two conformational states occupy distinct chemical spaces for ligand recognition; a high-affinity binder for the agonist pocket does not necessarily confer high affinity for the antagonist pocket, and vice versa. This orthogonality supports the need to train separate surrogate models for each state to capture their unique structure–docking-score relationships.

**Figure 3:**
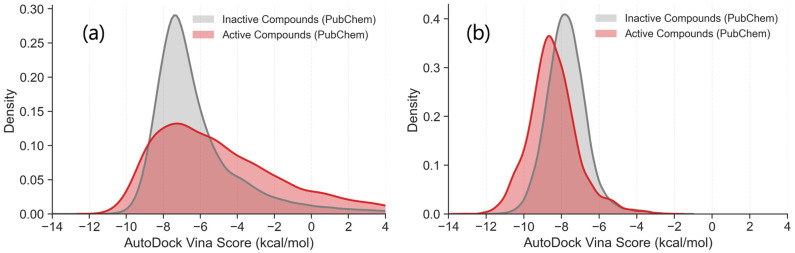
Validation of docking performance using PubChem bioactivity data. Kernel density estimation (KDE) plots comparing AutoDock Vina score distributions for known active (red) and inactive (gray) compounds. (**a**) The agonist conformation (1GWR) shows overlapping distributions, failing to discriminate active compounds due to steric constraints of the closed Helix 12. (**b**) The antagonist conformation (3ERT) exhibits a clear separation between active and inactive compounds, showing significantly more favorable docking scores for the former, confirming its suitability for identifying antagonist ligands.

This state-dependent selectivity is qualitatively illustrated by the docking performance of compounds annotated as active in PubChem ERα BioAssays (Figure 3). The antagonist conformation (3ERT) showed clearer separation between PubChem assay-annotated active and inactive compounds, with the former exhibiting a more favorable mean docking score of −8.44 kcal/mol. In contrast, the agonist conformation (1GWR) showed substantial overlap between the two groups, with active compounds exhibiting a weaker mean docking score of −4.77 kcal/mol. This apparent discrepancy likely reflects the non-state-specific and mechanistically heterogeneous nature of PubChem activity annotations, which may include agonists, antagonists, SERMs, partial agonists, and assay-dependent modulators. Accordingly, bulky or antagonist-like active compounds may be poorly accommodated within the closed agonist-bound 1GWR pocket, leading to less favorable, or even positive, docking scores despite their active annotations. Taken together, these observations are broadly consistent with, though do not independently establish, distinct ligand-recognition constraints. Because PubChem activity annotations are not resolved by mechanism of action, this comparison is presented as a qualitative observation rather than definitive evidence; the primary rationale for state-resolved surrogate modeling instead derives from the docking-score distributions and low cross-conformation correlation described above (Figure 2).

A plausible energy window of [−20, 10] kcal/mol was adopted a priori to flag numerical extremes. For the 1GWR dataset, 335,383 ligands passed basic ingestion, among which 4381 entries fell outside the plausible energy window and were flagged as outliers. For the 3ERT dataset, 332,005 ligands passed basic ingestion, with 43 outliers flagged. After applying all receptor-specific docking, conversion, deduplication, and quality-control (QC) filters—defined in Section 3.1 as structural sanitization/valence checks, restriction to a medicinal-chemistry element whitelist, and removal of salts/solvents, together with the docking-score outlier screening described above—326,057 ligands remained for 1GWR, and 328,279 ligands remained for 3ERT. The 1GWR score distribution had a median ΔG of −6.591 kcal/mol and a mean of −5.908 kcal/mol; using a threshold of ΔG ≤ −10.0 kcal/mol, 1433 ligands (0.44%) were designated as hits. For 3ERT, the score distribution showed a median ΔG of −7.436 kcal/mol and a mean of −7.270 kcal/mol, yielding 6135 hits (1.87%) at the same threshold. When hits were defined using the Vina threshold of ΔG ≤ −10.0 kcal/mol, 53.51% of 3ERT hits were within the top 1% of the Vina-ranked list. Taken together, these results indicate a left-shifted and tighter ΔG distribution for 3ERT relative to 1GWR. This is consistent with the effectively larger and altered cavity in the antagonist-like state, resulting from the displacement of H12, thereby allowing enhanced hydrophobic engagement. The stronger early concentration of threshold-defined hits in 3ERT underscores a more pronounced separation between likely binders and nonbinders in this open conformation, whereas the restricted pocket of 1GWR results in comparatively modest early enrichment.

### 2.2. Implementation and Architecture of the SurroDock Framework

To enable rapid virtual screening of large-scale compound libraries, SurroDock was implemented as an end-to-end deep learning framework for predicting AutoDock Vina docking scores from molecular features. The framework integrates data preprocessing, feature generation, model training, validation, and inference within a modular Python package built on TensorFlow. Although developed for Vina-score surrogate modeling in this study, its architecture is generalizable to other cheminformatics prediction tasks when appropriate training data are available.

The SurroDock workflow consists of three main stages: data processing, feature engineering, and model training (Figure S2). In the data-processing stage, raw molecular records are loaded, canonicalized, and sanitized to remove invalid structures. In the feature-engineering stage, multiple 2D molecular representations are generated, including Morgan fingerprints, MACCS keys, RDKit physicochemical descriptors, Vina-inspired scoring features, atom-pair fingerprints, and 2D pharmacophore fingerprints. These descriptors are deterministically assembled into high-dimensional feature vectors according to the selected model configuration. In the training stage, multilayer perceptron models are trained using memory-efficient streaming and mixed-precision computation, enabling stable learning on datasets containing hundreds of thousands of compounds.

To support extensibility and reproducibility, SurroDock uses a hierarchical package structure that separates user-facing command-line interfaces from backend modules for feature extraction, model construction, cross-validation, and inference (Figure S3). This design allows users to execute training, prediction, and fine-tuning workflows while enabling core algorithms and feature sets to be modified without changing the execution scripts. The detailed module organization is summarized in Table S1.

In addition to regression modeling, SurroDock provides practical utilities for large-scale screening workflows, including high-throughput score prediction, k-fold cross-validation, and automated reporting of performance metrics such as coefficient of determination (*R*^2^), root mean squared error (RMSE), and mean absolute error. This integrated implementation provides the technical foundation for the large-scale surrogate modeling experiments described in the following sections.

### 2.3. Evaluation of Cumulative Feature Combinations

Using the implemented SurroDock framework, a cumulative evaluation of molecular feature combinations was performed to identify descriptor sets that best balance predictive accuracy and computational efficiency. The analysis was performed using the NCI Open Database after receptor-specific docking and filtering of compounds with docking scores outside the predefined plausible energy window of −20 to 10 kcal/mol. The resulting datasets contained 231,616 compounds for 1GWR and 236,236 compounds for 3ERT.

**Figure 4:**
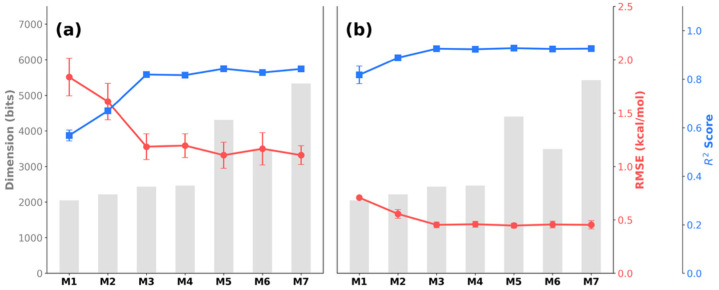
Performance evaluation of cumulative feature combinations on the 1GWR and 3ERT datasets. (**a**) 1GWR and (**b**) 3ERT results obtained using 5-fold scaffold-based cross-validation. Gray bars represent feature dimensionality (bits, left axis), while red circles and blue squares indicate mean RMSE (kcal/mol) and R^2^ values, respectively. Error bars denote the standard deviation across the five folds. The cumulative feature sets are defined as follows: M1, Morgan fingerprints; M2, M1 + MACCS keys; M3, M2 + RDKit physicochemical descriptors; M4, M3 + Vina-inspired docking-related descriptors; M5, M4 + atom-pair fingerprints; M6, M4 + 2D pharmacophore fingerprints; and M7, all feature classes combined.

Feature sets were constructed cumulatively to estimate the marginal contribution of each descriptor class. M1 used Morgan fingerprints; M2 added MACCS keys to M1; M3 added RDKit physicochemical descriptors to M2; M4 added Vina-inspired ligand descriptors to M3; M5 added atom-pair fingerprints to M4; M6 added 2D pharmacophore fingerprints to M4; and M7 combined all feature classes. Model performance was assessed using 5-fold scaffold-based cross-validation to evaluate generalization (Figure 4) and whole-dataset fitting evaluation to assess maximum fitting capacity (Figure S4).

The cross-validation results showed progressive improvement as complementary descriptors were added. For 1GWR, the baseline M1 model achieved an *R*^2^ of 0.568 and an RMSE of 1.838 kcal/mol. Adding structural keys and physicochemical descriptors substantially improved performance, with M3 reaching an *R*^2^ of 0.819 and an RMSE of 1.185 kcal/mol. The best cross-validation performance was obtained with M5, which achieved an *R*^2^ of 0.843 and an RMSE of 1.106 kcal/mol. A similar trend was observed for 3ERT, although the overall prediction task was easier: M3 already achieved high accuracy (*R*^2^ = 0.925, RMSE = 0.454 kcal/mol), while M5 provided a modest additional improvement (*R*^2^ = 0.928, RMSE = 0.447 kcal/mol).

Comparison with whole-dataset fitting performance revealed different generalization behavior between the two receptor states. For 1GWR, M5 achieved strong whole-dataset fitting (*R*^2^ = 0.980, RMSE = 0.393 kcal/mol), but its cross-validation performance was lower (*R*^2^ = 0.843, RMSE = 1.106 kcal/mol), suggesting partial overfitting or greater heterogeneity in the agonist-bound pocket. In contrast, 3ERT showed a smaller gap between whole-dataset fitting (*R*^2^ = 0.947, RMSE = 0.382 kcal/mol) and cross-validation performance (*R*^2^ = 0.928, RMSE = 0.447 kcal/mol), indicating a more stable generalization.

The trade-off between accuracy and computational cost was also evident. Although Vina-inspired docking-related descriptors in M4 added little predictive gain relative to M3 for 1GWR, they incurred negligible computational overhead. In contrast, 2D pharmacophore fingerprints in M6 substantially increased training time for 1GWR (419.6 s) relative to M3 (234.9 s) and M5 (219.7 s), without improving accuracy (*R*^2^ = 0.827). Taken together, M3 and M4 provide efficient configurations for large-scale screening when computational speed is prioritized, whereas M5 is preferable when maximal predictive accuracy is the primary objective, particularly for 1GWR (*R*^2^ = 0.843, RMSE = 1.106 kcal/mol), albeit with a greater risk of overfitting.

The interpretation of these results is limited by the sequential addition strategy, as the observed effects may depend on the order in which descriptors are introduced, owing to potential synergistic or compensatory effects among descriptor classes. Accordingly, the marginal gains reported above should be viewed as order-dependent estimates rather than definitive measures of each descriptor class’s intrinsic contribution.

### 2.4. Impact of Model Architecture on Predictive Performance

To determine an appropriate neural network configuration for SurroDock, this study evaluated the effects of network width (the number of neurons per hidden layer) and depth (the number of stacked hidden layers) on predictive performance using the M5 feature set. The baseline architecture, consisting of three hidden layers with 1024 units each, was compared with wider variants containing 2048 or 4096 units, a shallower variant containing two hidden layers, and deeper variants containing four hidden layers. Performance was assessed separately for the 1GWR and 3ERT datasets using 5-fold scaffold-based cross-validation with RMSE and *R*^2^ as the primary metrics.

As summarized in Table 1, neither decreasing nor increasing model complexity improves predictive performance. For 1GWR, the baseline architecture achieved the best overall performance, with an RMSE of 1.1057 kcal/mol and *R*^2^ = 0.8430. Increasing the width to 2048 or 4096 units slightly reduced performance, with *R*^2^ values of 0.8423 and 0.8368, respectively. Similarly, neither decreasing nor increasing the depth provides a consistent benefit, yielding *R*^2^ values of 0.8355–0.8417. These results suggest that the baseline model capacity was sufficient for the selected feature representation, whereas additional parameters provided little benefit and may have introduced mild overfitting or optimization inefficiency.

A similar pattern was observed for 3ERT. The baseline architecture achieved an RMSE of 0.4473 kcal/mol and an *R*^2^ of 0.9278, comparable to or better than those of the smaller and larger models. Although the 2048-unit model produced a slightly lower RMSE (0.4468 kcal/mol), its *R*^2^ was lower (*R*^2^ = 0.9255), and further increasing the width to 4096 units reduced performance (*R*^2^ = 0.9211). Deeper four-layer models also showed higher errors than the baseline, with RMSE values above 0.46 kcal/mol.

Overall, the three-layer, 1024-unit architecture provided the best balance between predictive accuracy and computational efficiency across both receptor states. This configuration was therefore adopted as the standard SurroDock architecture for subsequent experiments, although the two-layer configuration represents an equally valid, more lightweight alternative.

### 2.5. Impact of Morgan Fingerprint Radius and Bit Vector Length on Predictive Performance

This study systematically investigated the influence of the Morgan fingerprint radius and bit vector length on SurroDock performance to determine the appropriate structural resolution for molecular representation. The analysis was conducted on both the physicochemical-based M3 model and the final atom-pair augmented M5 model.

Contrary to the expectation that larger radii would consistently improve performance by capturing broader topological environments, compact radii generally produced better results (Table 2 and Table S2). For 1GWR using the M5 model, a radius of 2 with 2048 bits achieved the best performance, with an RMSE of 1.1057 kcal/mol and an *R*^2^ of 0.8430. Increasing the radius beyond 2 led to a gradual decline in performance; for example, radius 4 reduced *R*^2^ to 0.8345. A similar trend was observed for 3ERT, where the radius-2 setting with 2048 bits achieved strong performance (*R*^2^ = 0.9278, RMSE = 0.4473 kcal/mol), although the 4096-bit radius-2 setting produced a marginally higher *R*^2^ (*R*^2^ = 0.9280) with increased dimensionality.

The preference for compact Morgan radii in the M5 model is likely related to the complementary role of atom-pair fingerprints. Atom-pair fingerprints encode longer-range topological relationships and scaffold-level structural information, thereby complementing the local atomic environments captured by small-radius Morgan fingerprints. As a result, radius-2 Morgan fingerprints can provide high-resolution local substructure information without requiring encoding of more distant connectivity. Larger Morgan radii may therefore introduce redundant or sparse features when atom-pair descriptors are already included.

The comparison between 2048-bit and 4096-bit representations further indicated diminishing returns with increasing dimensionality. Although 4096-bit Morgan fingerprints provided marginal improvements in selected cases, such as M3 for 1GWR and M5 for 3ERT, these gains were small and did not consistently outweigh the additional memory and computational cost. Therefore, a radius-2, 2048-bit Morgan fingerprint configuration was selected as the standard representation for SurroDock. This choice provided a practical balance between predictive accuracy, computational efficiency, and compatibility with the complementary atom-pair descriptors used in M5.

### 2.6. Effects of Data Scale and Composition on SurroDock Generalization

The primary objective of SurroDock is to prioritize top-ranked docking candidates from large chemical libraries while maintaining reliable generalization beyond the training set. Because docking-score surrogate performance depends on both dataset size and chemical-space coverage, this section evaluates how data scale and composition affect model robustness. Three complementary analyses were performed: (i) learning-curve analysis using subsets of the NCI Open library, (ii) comparison of single-source and hybrid training datasets, and (iii) integrated random sampling from a combined pool of BindingDB, PubChem, and NCI Open compounds. Particular attention was given to externally held-out validation and to compounds with favorable reference Vina scores (∆G_Vina_ < −8.0 kcal/mol), which are most relevant for virtual-screening prioritization. All experiments used the M5 feature set with Morgan radius 2 and 2048-bit fingerprints.

#### 2.6.1. Data-Scale Dependence of Generalization and Overfitting

To quantitatively estimate the minimum sample size required for robust generalization, subsets of the NCI Open library were used to analyze the divergence between training performance and predictive accuracy, hereafter referred to as the generalization gap, across varying dataset sizes ranging from 5k to 200k compounds. This analysis was designed to identify the data-scale threshold at which the model transitions from overfitting-dominated behavior to stable generalization. Because the NCI Open library contains chemically diverse compounds, it provides broad coverage of ligand chemical space and is suitable for evaluating scale-dependent learning behavior.

**Figure 5:**
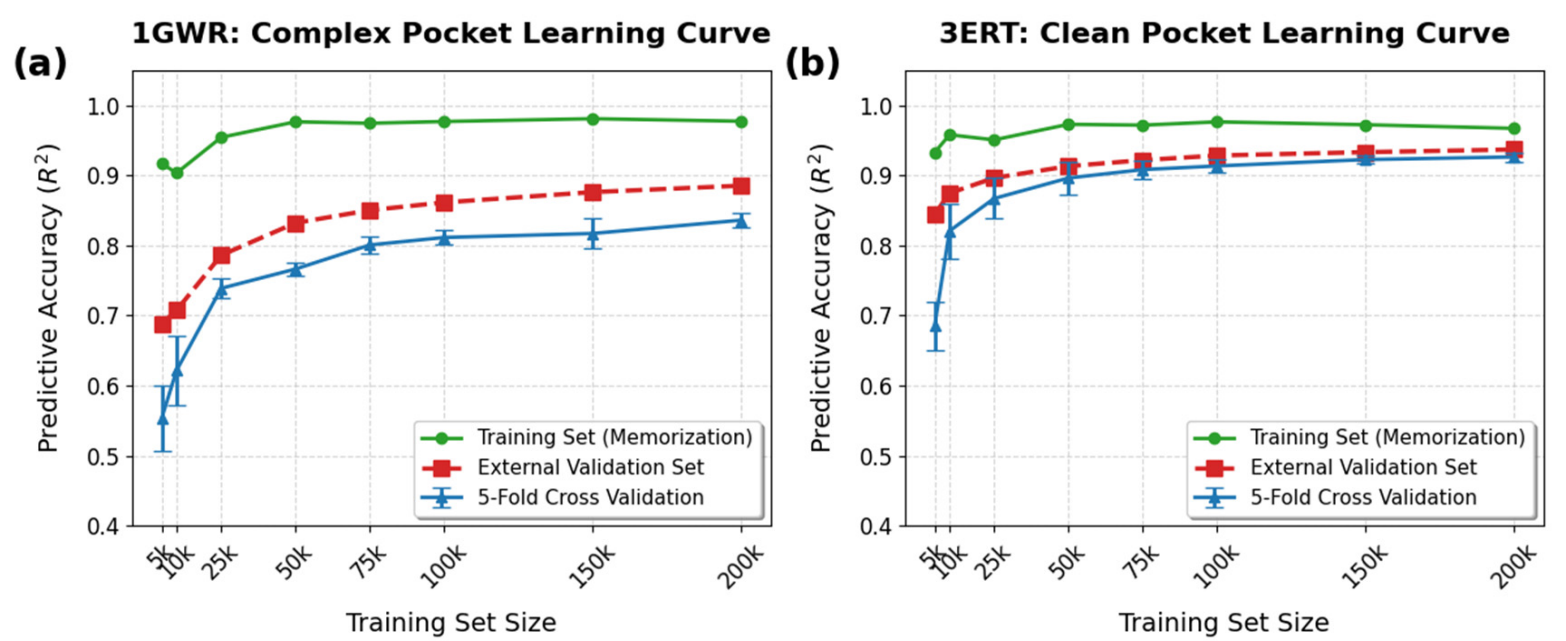
Learning curves of SurroDock as a function of training-set size for two binding pockets. Predictive accuracy, measured by *R*^2^, is reported for the training set (green), external validation set (red), and 5-fold cross-validation (blue) across training-set sizes ranging from 5k to 200k compounds. (**a**) The 1GWR model exhibits a large generalization gap in the low-data regime, which progressively narrows as the dataset size increases, indicating that larger-scale training is required to stabilize generalization. (**b**) The 3ERT model shows rapid gains at smaller dataset sizes and approaches a performance plateau around 100k–150k compounds, reflecting higher data efficiency. Error bars denote the standard deviation across cross-validation folds.

Learning-curve analyses using NCI Open subsets showed clear scale-dependent gains. Additionally, as illustrated in Figure 5, the data requirements depended on the target pocket’s structural characteristics. For 1GWR (Figure 5a), a severe generalization gap was observed in the low-data regime of 5k–25k compounds, with cross-validation and external-validation performance remaining substantially lower than training performance. At the smallest scale, cross-validation performance remained below *R*^2^ = 0.6, indicating a tendency toward memorization rather than the learning of generalizable structure–score relationships. However, this gap narrowed monotonically as the data scale increased beyond approximately 100k compounds, leading to more stable generalization performance.

In contrast, the model showed higher data efficiency for 3ERT (Figure 5b), with validation performance improving rapidly even at smaller dataset sizes, particularly at approximately 50k compounds. Performance then plateaued in the 100k–150k range, with additional data yielding only limited marginal gains. Collectively, these results suggest that smaller datasets may be sufficient for relatively data-efficient targets. In contrast, robust surrogate modeling across diverse and structurally complex binding pockets requires a training set of at least 100,000 compounds to mitigate overfitting effectively. Accordingly, this threshold was adopted as the baseline scale for subsequent experiments, allowing data-composition effects to be evaluated with reduced interference from data-scarcity effects.

#### 2.6.2. Synergistic Effect of Data Composition

The PubChem ERα Active/Inactive dataset comprises experimentally annotated bioactivity records and thus provides target-relevant chemical information. However, because these data originate from assay-specific experimental collections, their chemical diversity may be narrower than that of broader screening libraries, potentially introducing dataset bias and limiting external transferability. This limitation was evident for the 1GWR target. Although the PubChem-only model achieved strong internal performance, with *R*^2^ = 0.981 on the training set and *R*^2^ = 0.835 in cross-validation, it generalized poorly to the held-out validation set, yielding *R*^2^ = 0.495 and RMSE = 1.977 kcal/mol.

To alleviate this chemical-space restriction while retaining target relevance, hybrid datasets were constructed by augmenting PubChem compounds with increasing amounts of chemically diverse NCI Open compounds, forming mixtures ranging from 150k compounds at a 1:0.5 ratio to 300k compounds at a 1:2 ratio. External generalization improved substantially with this expansion. For 1GWR, *R*^2^ (HOV) increased to 0.776 for Mix-150k and reached 0.818 with an RMSE (HOV) of 1.238 at the balanced 200k scale, corresponding approximately to a 1:1 mixture. Performance gains were modest beyond this point in the 250k–300k range, indicating diminishing returns after the balanced mixture. These results suggest that combining PubChem-derived target-relevant information with the broader chemical diversity of the NCI Open library reduces dataset bias and improves generalization for structurally demanding targets.

Building on these controlled mixtures, robustness was further assessed under unbiased integrated sampling by merging all available docking-derived training candidates into a single compound pool comprising BindingDB, PubChem ERα Active/Inactive, and NCI Open compounds. Randomized training subsets of 150k, 200k, 225k, and 250k compounds were then drawn from this integrated pool, with the remaining compounds used as the held-out validation set. As shown in Table 3, the integrated approach outperformed single-source models across settings. For 1GWR, Unified-200k achieved an *R*^2^ (HOV) of 0.881 and an RMSE (HOV) of 0.943, substantially improving upon both the target-focused PubChem-only model with an *R*^2^ (HOV) of 0.495 and the diversity-focused NCI Open-only model with an *R*^2^ (HOV) of 0.753. Increasing the training scale to 250k yielded only marginal gains, with less than 1.2 percentage-point absolute improvement in *R*^2^ (HOV), while incurring greater computational cost. Thus, 200k compounds represented the most cost-effective scale in the integrated-sampling setting. A similar plateau was observed for 3ERT, where external performance stabilized at approximately *R*^2^ (HOV) = 0.93 across integrated scales.

In addition to global regression metrics such as RMSE and *R*^2^, ranking-oriented performance was assessed using the concordance index (CI) and top-1% recovery, reported as Recall@1%. CI measures the probability that the model preserves the correct ordering for a randomly selected pair of compounds, whereas Recall@1% was defined as the fraction of Vina top-1% compounds recovered in the SurroDock-predicted top 1%, directly reflecting top-hit recovery in virtual-screening prioritization. For 3ERT, the advantage of integrated sampling was particularly clear in external ranking metrics: the NCI Open-only model achieved CI (HOV) = 0.867 and Recall@1% (HOV) = 0.242, whereas Unified-200k improved these values to CI (HOV) = 0.920 and Recall@1% (HOV) = 0.606. The PubChem-only model showed a marked internal-to-external drop in top-hit recovery, with Recall@1% = 0.829 in cross-validation but only 0.342 on the held-out validation set. This result indicates that strong internal performance does not necessarily translate into robust external top-hit retrieval, consistent with chemical-space bias in single-source training and its mitigation through data integration.

Finally, because the practical objective of virtual screening is to prioritize top-ranked docking candidates, model behavior in the favorable-docking-score region is particularly important. In this region, defined as the HOV-H subset with reference Vina scores satisfying ∆G_Vina_ < −8.0 kcal/mol, global error metrics such as RMSE can be less discriminative because of the reduced score variance among top-ranked compounds. Ranking fidelity is therefore more directly captured by metrics such as Pearson correlation, CI, and Recall@1%. The integrated-sampling model, Unified-200k, maintained robust correlations in this favorable-score window, with Pearson r = 0.613 for 1GWR HOV-H and r = 0.776 for 3ERT HOV-H. The accompanying improvements in CI and Recall@1% further support that SurroDock enhances both overall ranking consistency and top-hit recovery in the favorable-docking-score region most relevant to practical virtual screening.

### 2.7. Core Regression Performance and Distributional Fidelity

To validate the fundamental predictive capability of SurroDock, the M5 model trained on the Unified-200k dataset was selected for detailed regression analysis. Evaluation was conducted on both the training and held-out validation sets, comprising approximately 129k compounds for 1GWR and 134k compounds for 3ERT. Model performance was assessed across two distinct binding environments: the agonist-bound pocket of 1GWR and the antagonist-bound pocket of 3ERT.

**Figure 6:**
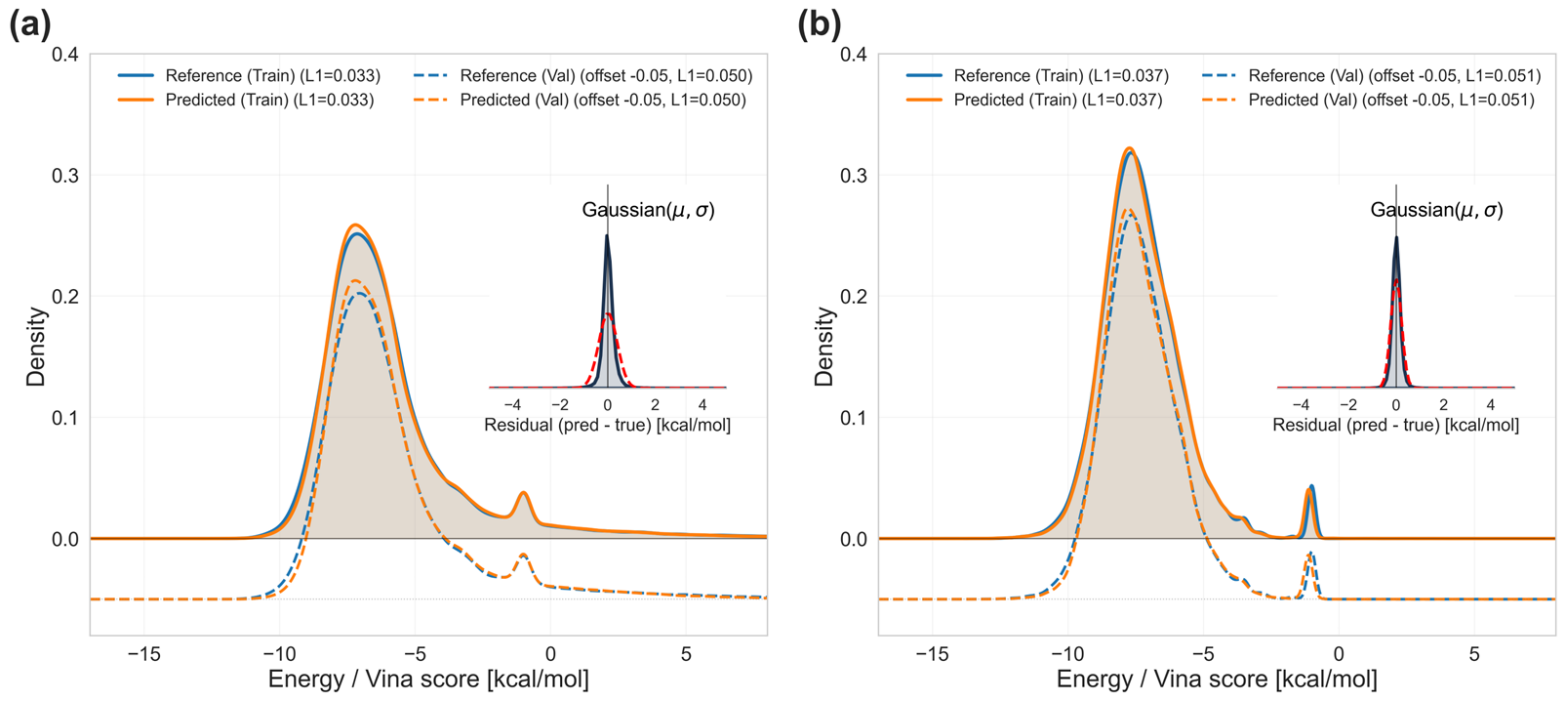
Comparison of reference and SurroDock-predicted docking-score distributions for 1GWR and 3ERT. Kernel density estimates, KDEs, are shown for (**a**) 1GWR and (**b**) 3ERT. Solid curves correspond to the training set, and dashed curves correspond to the held-out validation (HOV) set, shifted vertically by −0.05 for visual clarity. Blue curves denote reference AutoDock Vina scores, and orange curves denote SurroDock-predicted scores. Insets show residual distributions, defined as y_pred_ − y_true_, with the histogram in gray and the corresponding Gaussian fit overlaid as a red curve.

Figure 6 compares kernel density estimates, KDEs, of the reference Vina and SurroDock-predicted scores. Beyond point-wise accuracy metrics, reproducing the global statistical distribution of docking scores is important for reliable virtual-screening prioritization. As shown in the plots, the M5 model exhibited strong distributional fidelity for both the more challenging 1GWR target (Figure 6a) and the 3ERT target (Figure 6b). The predicted distributions closely overlapped with the reference distributions, capturing not only the main score peaks but also secondary modes, such as the minor mode near −1 kcal/mol in 3ERT. The consistency between the training and held-out validation distributions further supports the model’s robustness. In particular, the validation KDEs showed no substantial degradation in distributional shape, suggesting that the model captured the statistical structure of the Vina scoring function rather than merely memorizing training samples. Moreover, agreement in the favorable-score tail, particularly for Vina scores below −9 kcal/mol, indicates that SurroDock preserves the score statistics of top-ranked docking candidates.

**Figure 7:**
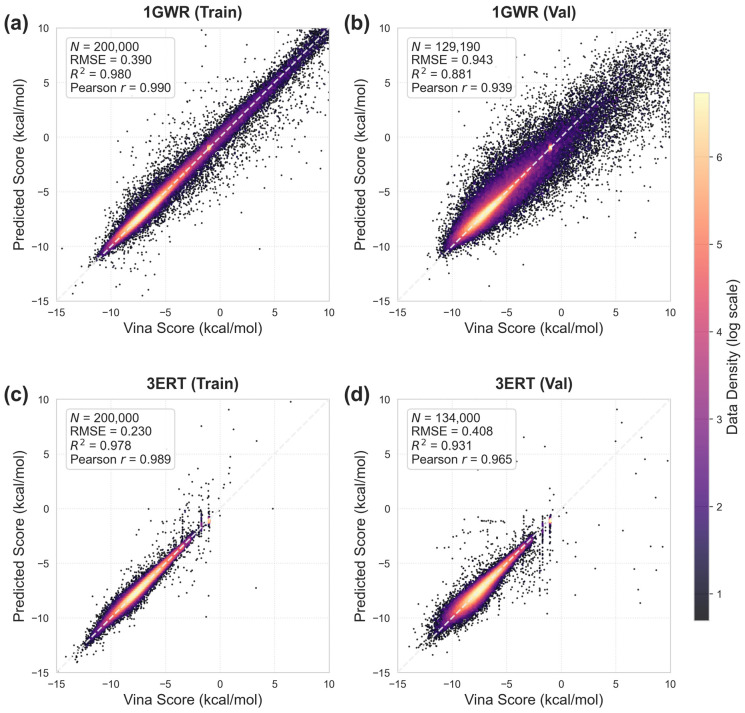
Correlation between SurroDock-predicted Vina and reference Vina scores on the 1GWR and 3ERT datasets. Density scatter plots (log-scaled point density) summarize agreement between predicted scores (y-axis) and Vina scores (x-axis) for 1GWR ((**a**) train, (**b**) held-out validation (HOV)) and 3ERT ((**c**) train, (**d**) held-out validation (HOV)). The white dashed diagonal denotes perfect agreement, y = x. Each panel reports N, RMSE, *R*^2^, and Pearson correlation coefficient r.

Figure 7 shows density scatter plots comparing SurroDock-predicted scores with reference Vina scores. For 1GWR, the model achieved strong agreement on the training set (Figure 7a), with *R*^2^ = 0.980, RMSE = 0.390 kcal/mol, and Pearson *r* = 0.990. On the held-out validation set (Figure 7b), performance decreased but remained robust, with *R*^2^ = 0.881, RMSE = 0.943 kcal/mol, and Pearson *r* = 0.939. This generalization gap likely reflects the more complex conformational and scoring landscape of the 1GWR agonist-bound pocket. Nevertheless, the high Pearson correlation in validation indicates that the linear relationship between the predicted and reference Vina scores was largely preserved.

For 3ERT (Figure 7c,d), the model maintained consistently high performance on both the training set and the held-out validation set. The training set showed *R*^2^ = 0.978, RMSE = 0.230 kcal/mol, and Pearson *r* = 0.989, whereas the held-out validation set showed *R*^2^ = 0.931, RMSE = 0.408 kcal/mol, and Pearson *r* = 0.965. These results indicate limited overfitting in the 3ERT antagonist-bound pocket and demonstrate that SurroDock can retain both high pointwise accuracy and strong ranking consistency in external validation (see Supplementary Figures S5 and S6 for complementary calibration and residual-distribution diagnostics).

### 2.8. Virtual Screening Efficiency: Early Enrichment

**Figure 8:**
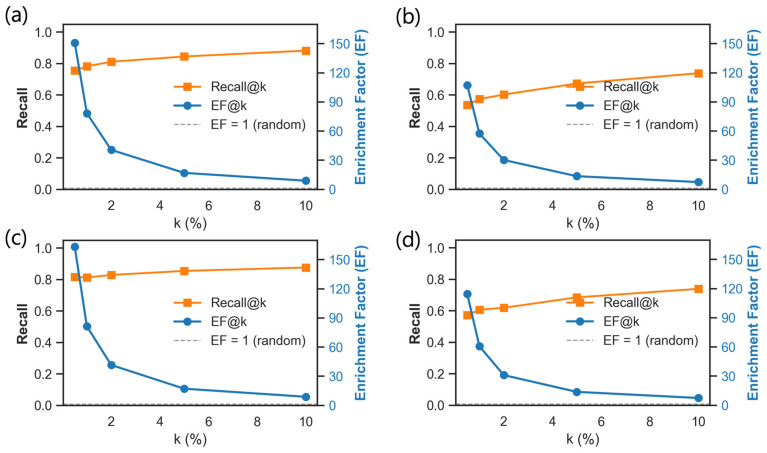
Top-k ranking performance and early enrichment for 1GWR and 3ERT. Recall@k and EF@k are reported at k values of 0.5%, 1%, 2%, 5%, and 10% for (**a**) 1GWR training, (**b**) 1GWR HOV, (**c**) 3ERT training, and (**d**) 3ERT HOV sets. Reference and predicted top-k sets were defined as the top k% ranked by Vina and SurroDock scores, respectively. Recall@k denotes the fraction of reference Vina top-k% compounds recovered in the SurroDock-predicted top-k% set. The orange squares denote Recall@k, the blue circles denote EF@k, and the dashed gray line indicates random enrichment, EF = 1.

The goal of SurroDock is to accelerate large-scale virtual screening by prioritizing compounds with favorable, low Vina scores. To quantify early ranking utility, top-k recovery and enrichment were evaluated using Recall@k and EF@k across multiple k values, expressed as percentages of the screened library. The reference top-k set was defined as the compounds ranked within the top k% by Vina score, whereas the predicted top-k set was defined as the compounds ranked within the top k% by SurroDock score. Recall@k was computed as the fraction of reference Vina top-k% compounds recovered in the SurroDock-predicted top-k% set, and EF@k was calculated as Recall@k divided by k/100. Figure 8 summarizes the early-enrichment performance. For 1GWR (Figure 8a,b), SurroDock showed strong early enrichment despite the greater complexity of the 1GWR pocket. On the held-out validation set at k = 1%, agreement with the reference Vina top-1% set reached Recall@1% = 0.573, corresponding to EF@1% = 57.3, far above the random baseline of EF = 1. For 3ERT (Figure 8c,d), early enrichment was also strong, with Recall@1% = 0.606 and EF@1% = 60.6 on the held-out validation set. Overall, the strong enrichment observed at small k values indicates that SurroDock can serve as an effective pre-filter for triaging ultra-large compound libraries while concentrating a substantial fraction of top-ranked docking candidates within a small selection budget.

In addition to ranking-based enrichment, the computational cost of SurroDock relative to direct docking is another key factor determining its practical value. Table 4 summarizes this comparison, normalized to 200,000 compounds using the empirically measured mean Vina runtimes on the NCI Open dataset (18.13 s/compound for 1GWR and 55.77 s/compound for 3ERT). Whereas Vina required 1007–3098 CPU-core-hours to dock this many compounds, SurroDock needed only 16–24 min of one-time GPU training, after which the same 200,000 compounds could be scored in about 10 s.

The advantage becomes more apparent as library size increases. Docking 10 million compounds directly with Vina would require roughly 50,000–155,000 CPU-core-hours, and several thousand CPU-core-days, which is infeasible with typical institutional resources. SurroDock, by contrast, can pre-screen the same library in about 8–9 min once trained, and re-docking only the top-ranked 1% (about 100,000 compounds) with AutoDock Vina for confirmation requires just 500–1550 CPU-core-hours—roughly two orders of magnitude less than exhaustive docking. In this way, SurroDock changes what is computationally achievable, turning a months-long docking campaign into one completed in a fraction of that time.

### 2.9. Applicability Domain and Robustness

A common concern with machine learning-based surrogate scoring functions is whether prediction accuracy is retained for compounds that are chemically dissimilar to the training distribution. To assess this applicability-domain behavior, the relationship between prediction error and chemical similarity to the training set was analyzed. Chemical similarity was quantified as the mean Tanimoto similarity to the 25 nearest neighbors in the training set, and prediction error was measured as the absolute residual, |y_pred_ − y_true_|.

**Figure 9:**
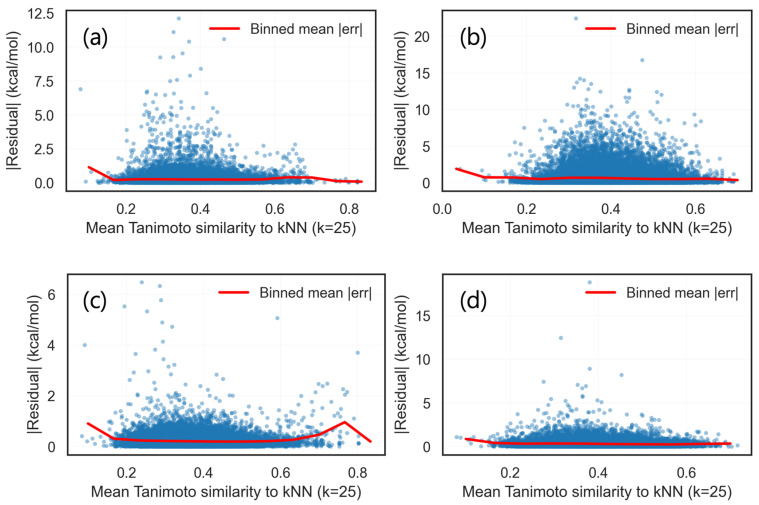
Similarity–error analysis for applicability-domain assessment. Scatter plots show absolute error $|y_{pred}-y_{true}|$ (y-axis) versus the mean Tanimoto similarity to the k=25 nearest neighbors in the training set (x-axis). Panels correspond to 1GWR ((**a**) train, (**b**) held-out validation) and 3ERT ((**c**) train, (**d**) held-out validation). The red line shows the binned mean absolute error, illustrating only a weak dependence of error on similarity, including in low-similarity regions.

Figure 9 plots the absolute residual against the mean k-nearest-neighbor Tanimoto similarity. For 1GWR (Figure 9a,b), the binned mean absolute error varied only mildly with similarity in both the training and held-out validation sets. Although scattered high-error outliers were observed, the binned trend did not show a systematic increase in error at lower similarity. For 3ERT (Figure 9c,d), the mean absolute error likewise did not sharply increase in the low-similarity regime, including compounds with a mean Tanimoto similarity below approximately 0.3. Mild variation was observed across the similarity range, but the overall trend remained relatively flat.

These results suggest that SurroDock performance is not strongly dependent on the presence of close analogs in the training set. Instead, the weak similarity–error dependence supports the model’s ability to capture generalizable structure–score relationships across chemically diverse compounds, thereby extending its practical applicability domain beyond highly similar training-set neighbors (see Supplementary Figure S7 and Table S3 for a complementary similarity-stratified conformal prediction analysis).

Additionally, in the intended deployment scenario, SurroDock is used to score an ultra-large compound library, with a selection margin above the target screening fraction subsequently re-docked prior to final hit selection; every advanced compound is thus re-evaluated by actual docking rather than relying solely on the surrogate prediction. Under this design, the key practical question is how large a selection margin is needed to reliably recover true top-ranked compounds. A Top-K screening degradation analysis on the hold-out validation set indicates that a margin on the order of 5- to 10-fold above the target fraction recovers the large majority of true top-ranked compounds (Supplementary Figure S8).

## 3. Materials and Methods

### 3.1. Data Sources and Curation

Small-molecule libraries were assembled from three public sources. A general-purpose chemical library was obtained as SDF archives from the NCI Open Database (Release 4 May 2012; Developmental Therapeutics Program, National Cancer Institute) through the NCI CADD portal [20], yielding 265,242 unique entries. This dataset is referred to hereafter as the NCI Open library. An ERα (UniProt P03372)–focused set was compiled from PubChem BioAssays [4,21] using assay-reported activity outcome annotations to label compounds as active (7923) or inactive (90,291). Records were exported as SDF and SMILES in CSV. Experimentally curated interaction data were also retrieved from BindingDB [22] using ERα as the protein query, yielding 9528 compounds. All small-molecule entries underwent a standardized curation workflow prior to 3D preparation or docking, in accordance with the established cheminformatics practices [14]. Salts and solvents were removed, retaining the largest organic component per record, and aromaticity was assigned under a consistent convention. Quality-control filters excluded entries failing sanitization or valence checks and restricted elements to a standard medicinal-chemistry whitelist (H, C, N, O, F, P, S, Cl, Br, I, B, Si). To support reproducibility, each record was tagged with its source (NCI Open, PubChem-ERα, BindingDB) and original identifiers (e.g., NSC, CID, BindingDB IDs).

Following curation, receptor-specific docking and post-processing filters were applied independently to the two ERα conformations (1GWR and 3ERT). Because docking preparation and QC were performed independently for each receptor conformation, the final retained counts differed slightly between 1GWR and 3ERT. Source-specific retention after docking and QC was, for 1GWR and 3ERT, respectively: 4561 and 4694 compounds from BindingDB; 7665 and 7721 PubChem ERα actives and 89,908 and 90,043 inactives; and 231,616 and 236,236 compounds from the NCI Open Database. Cross-source duplicate records were identified using RDKit canonical SMILES, while retaining source provenance and original identifiers for auditability. For receptor-specific analyses, docking and QC status were tracked independently for each ERα conformation.

### 3.2. Target Structures and Protein Preparation

ERα LBD structures were obtained from the RCSB Protein Data Bank [23]. Two crystallographic conformations were selected as primary receptors: the agonist-bound form (PDB ID: 1GWR) [24] and the SERM/antagonist (4-Hydroxytamoxifen)-bound form (PDB ID: 3ERT) [25]. Crystallographic water molecules were removed, and structures were visually inspected using PyMOL, v3.1.1 (Schrödinger, LLC, New York, NY, USA) [26] to confirm the ligand-binding region and the absence of metal ions or cofactors. Receptors were converted to PDBQT format using AutoDockTools, v1.5.6 (Molecular Graphics Laboratory, The Scripps Research Institute, La Jolla, CA, USA) with default settings [27], including Gasteiger charge assignment [28]. For each receptor, the docking search space was centered on the co-crystallized ligand. The box size along each Cartesian axis was defined as the ligand’s axis-aligned extent plus 15 Å. Docking and subsequent analyses were performed independently for each receptor conformation to maintain structural state specificity.

### 3.3. Ligand Processing

Ligand structures from the NCI Open Database, PubChem BioAssays, and BindingDB were prepared using a standardized workflow prior to docking. For ligands from all three sources, PubChem CIDs were used whenever available to retrieve corresponding three-dimensional SDF records from PubChem. Compounds without retrievable PubChem 3D SDF records were excluded from docking preparation. Stereochemistry and conformers were retained as provided by PubChem, and no additional 3D conformer generation or force-field energy minimization was performed prior to docking. Following the source-level curation and quality-control steps described in Section 3.1, ligands were converted to MOL2 format using Open Babel, v3.1.1 [29]. During this conversion, the protonation states of the ligands were adjusted to pH 7.4 using the -p 7.4 option in Open Babel. The resulting structures were subsequently prepared as PDBQT files using AutoDockTools [27], with Gasteiger charges [28]. Non-polar hydrogens were merged into their parent heavy atoms, while polar hydrogens were retained explicitly to preserve hydrogen-bond-donor geometry. Prior to format conversion, structures were checked using RDKit, v2025.03.5 [30], sanitization, and valence validation. Compounds containing metals, inorganic species, or polymers were excluded. Ligands failing the MOL2 or PDBQT conversion were excluded from downstream docking.

### 3.4. Docking Protocol

AutoDock Vina, v1.2.5 (Center for Computational Structural Biology, The Scripps Research Institute, La Jolla, CA, USA) [8,9] was used to generate state-resolved docking-score datasets for ERα in two conformational states using default parameters. Prior to large-scale docking, the protocol was validated by redocking each co-crystallized ligand into its cognate receptor structure, estradiol into 1GWR and 4-hydroxytamoxifen into 3ERT and assessing both the docking score and pose agreement using heavy-atom RMSD relative to the crystallographic pose. After validation, the prepared compound library was docked against both receptor states to obtain paired, state-specific docking energies. The best-scoring pose, corresponding to the lowest predicted binding energy, was used as the docking label. No post-docking energy minimization was applied beyond Vina’s internal refinement. Docking runs exceeding 5 min for a given ligand–receptor pair were terminated and excluded from downstream model training and evaluation to manage computational cost. This exclusion criterion was applied uniformly across receptor states, and the corresponding docking/QC status was recorded for auditability. To flag numerical extremes and stabilize downstream learning, docking outputs were screened using a plausible energy window of −20 to 10 kcal/mol; entries outside this range were marked as outliers and excluded from model training and evaluation. Threshold-based virtual-screening analyses used predefined docking-score cutoffs where applicable, whereas rank-based early-enrichment analyses were based on top-k Vina and SurroDock rankings. State-dependent enrichment behavior was analyzed separately for 1GWR and 3ERT. All docking calculations were performed on a single workstation equipped with an Intel Core i9-9900K CPU (Intel Corporation, Santa Clara, CA, USA), 32 GB RAM, and an NVIDIA GeForce RTX 2080 Ti, 11 GB (NVIDIA Corporation, Santa Clara, CA, USA) GPU running Ubuntu 24.04.1 LTS (Canonical Ltd., London, UK).

### 3.5. SurroDock Feature Construction (M1–M7) and Preprocessing

SurroDock, a neural network surrogate model for approximating AutoDock Vina docking scores from two-dimensional molecular representations, was implemented in Python, v3.12 (Python Software Foundation, Wilmington, DE, USA). Deep learning operations were performed using TensorFlow, v2.19.0 (Google LLC, Mountain View, CA, USA) and Keras, v3.1.11 (Google LLC, Mountain View, CA, USA) [31,32]. Cheminformatics tasks were handled using RDKit, v2025.03.5 [30], with numerical operations supported by NumPy, v2.5.0 and scikit-learn, v1.7.1 [33]. Input records from each source, consisting of SMILES strings and continuous docking-energy targets, were merged and harmonized prior to featurization. Duplicate records were removed using RDKit canonical SMILES. SMILES validity was verified using RDKit parsing and sanitization, and invalid entries were discarded. Each ligand was featurized from its curated SMILES string using RDKit-based molecular fingerprints and descriptors. The evaluated feature classes included Morgan fingerprints [34], MACCS keys [35], RDKit physicochemical descriptors [30], ligand-only descriptors motivated by Vina scoring terms, atom-pair fingerprints [36], and two-dimensional pharmacophore, Pharm2D, fingerprints [37]. The Vina-motivated descriptors were designed to capture ligand properties related to torsional flexibility, hydrogen-bonding capacity, heteroatom composition, ring and aromaticity patterns, formal charge, polarity, hydrophobicity, and conformational flexibility. Seven cumulative feature configurations were evaluated to quantify the marginal utility of each representation. M1 consisted of Morgan fingerprints alone. M2 added MACCS keys to M1. M3 added RDKit physicochemical descriptors to M2. M4 added the ligand-only Vina-motivated descriptors to M3. M5 augmented M4 with atom-pair fingerprints. M6 augmented M4 with Pharm2D fingerprints. M7 combined all feature classes included in M1–M6. High-dimensional feature vectors were assembled deterministically according to the selected feature configuration. Continuous descriptors were standardized using statistics estimated only from the training data, whereas binary fingerprint features were retained as binary inputs. All preprocessing steps, including descriptor scaling, were fit exclusively within the training fold or split and then applied to the corresponding validation or test data to prevent data leakage. The M1–M7 feature-contribution experiments were performed using the NCI Open Database release 4 May 2012. After receptor-specific docking and exclusion of numerical extremes outside the predefined plausible energy window of −20 to 10 kcal/mol, the feature-evaluation datasets contained 231,616 compounds for 1GWR and 236,236 compounds for 3ERT.

### 3.6. Model Architecture and Training

SurroDock used a feed-forward multilayer perceptron consisting of three hidden layers with 1024 units per layer and swish activations. Each hidden layer was followed by batch normalization and dropout, with a dropout rate of 0.3. The output layer consisted of a single linear unit predicting docking energy, with outputs computed in float32 for numerical stability.

Models were trained to minimize mean squared error using the Adam optimizer [38] with a learning rate of 1 × 10^−4^, a batch size of 128, and gradient clipping (clipnorm = 1.0). Early stopping, with patience = 20, best-weight restoration, and validation-loss checkpointing were used during training. Within each training run, 10% of the training data was reserved as an internal validation subset for early stopping and checkpoint selection. Mixed-precision training was enabled on NVIDIA GPUs [39]. Model training and inference were performed on the same workstation described in Section 3.2.

### 3.7. Validation and Evaluation

Generalization was assessed using 5-fold cross-validation with scaffold-based splits derived from Bemis–Murcko scaffolds [40] to reduce scaffold leakage and evaluate generalization across chemotypes. Preprocessing was fit exclusively on training folds and then applied to validation or test folds to prevent data leakage. Y-scrambling validation (*n* = 10) was performed by permuting the target labels while preserving the scaffold splits [41,42]. Regression performance was evaluated using mean absolute error, RMSE, *R*^2^, Pearson correlation (r), and Spearman’s rank correlation (ρ) [43,44]. Calibration was assessed using linear regression of predicted versus true docking scores.

Ranking and virtual-screening performance were evaluated using Spearman’s ρ, Recall@k%, and enrichment factor (EF@k%). Recall@k% was defined as the fraction of reference Vina top-k% compounds recovered within the SurroDock-predicted top k% set:

$$Recall@k\%\;=\;\frac{\left|{TopK}_{Vina}\cap {TopK}_{SurroDock}\right|}{\left|{TopK}_{Vina}\right|}$$

where TopK_Vina_ and TopK_SurroDock_ denote the sets of top-k% compounds ranked by vina docking score and by SurroDock-predicted score, respectively. Because both sets had identical sizes, Recall@k% was equivalent to the top-k% overlap fraction. Enrichment factor at k% (EF@k%) was calculated as Recall@k% divided by the expected recall under random selection (k/100), representing the fold-enrichment achieved over random screening:

$$\mathrm{EF}@k\%=\frac{\mathrm{Recall}@k\%}{k/100}$$

Threshold-based hit analyses used predefined Vina-score cutoffs, including −7.0 and −10.0 kcal/mol, where applicable, whereas rank-based analyses compared top-k compound sets defined by Vina and SurroDock rankings.

Each cumulative feature configuration was evaluated using two complementary strategies. First, 5-fold cross-validation was used to estimate generalization performance, with RMSE and *R*^2^ reported as the primary regression metrics. Second, a whole-dataset fitting evaluation was performed to assess the maximum fitting capacity of each feature set and to diagnose potential overfitting. The whole-dataset evaluation was not used to estimate prospective generalization but served as a reference for comparing fitting capacity with cross-validation performance. Wall-clock training time was recorded under identical computational settings to compare the efficiency of different feature configurations.

## 4. Conclusions

This study introduced SurroDock, a lightweight deep-learning surrogate designed to mitigate the docking-time bottleneck in ultra-large SBVS by approximating AutoDock Vina scores from low-cost molecular representations. Using ERα as a testbed across two binding environments, the agonist-bound pocket of 1GWR and the antagonist-bound pocket of 3ERT, a large protocol-consistent docking dataset was constructed, and a stability-hardened multilayer perceptron was trained on concatenated two-dimensional features, including Morgan fingerprints, MACCS keys, RDKit physicochemical descriptors, Vina-inspired ligand descriptors, atom-pair fingerprints, and 2D pharmacophore fingerprints.

Across held-out validation sets, SurroDock achieved strong agreement with reference Vina scores, with particularly high performance for 3ERT and robust generalization for the more challenging 1GWR setting. SurroDock reproduced not only point-wise accuracy but also the global score distribution and favorable-score tail behavior associated with top-ranked docking candidates, both of which are critical for screening applications. Early enrichment analyses further showed that SurroDock can concentrate a substantial fraction of top-ranked docking candidates within a small selection budget, supporting its use as an efficient pre-filter for library contraction prior to full docking.

Overall, SurroDock should be viewed not as a replacement for conventional docking, but as a practical pre-docking prioritization layer that enables docking resources to be focused on the most promising compounds and accelerates iterative design–test cycles. Because the framework is reproducible and retrainable given a target structure and docking protocol, it provides a scalable route for deploying target- and state-specific surrogates in high-throughput screening workflows. A trained SurroDock model is receptor- and protocol-specific and is not directly transferable to a different target, conformation, or docking protocol without retraining; what is transferable is the framework itself rather than the trained weights.

Several limitations should also be noted. SurroDock is trained to approximate Vina scores and therefore inherits the assumptions, noise, and biases of the underlying docking protocol; it does not directly predict experimental binding affinities. Because SurroDock is designed to emulate this specific docking protocol rather than to independently model binding thermodynamics, reproducing known scoring characteristics of AutoDock Vina—including its sensitivity to ligand size and hydrophobicity—is an anticipated consequence of faithful surrogate modeling, and any resulting bias in prioritized candidates is expected to be attenuated by the confirmatory re-docking step included in the proposed workflow. In addition, reliance on 2D ligand descriptors cannot fully capture the 3D geometric, protonation, and tautomeric effects that influence docking outcomes, leading to larger errors in specific chemical regions and rare outliers. Finally, applicability-domain analyses based on similarity–error relationships are diagnostic rather than definitive. Furthermore, the present validation is retrospective, comparing SurroDock’s predicted rankings against Vina rankings generated under the same docking protocol used for training; independent benchmarking against state-specific known actives/decoys and prospective experimental confirmation were not performed within the scope of this study. Therefore, conservative applicability-domain thresholds and selective confirmatory docking remain advisable when operating in low-similarity or high-uncertainty regimes.

SurroDock offers a substantial practical computational advantage: GPU-based inference reduces per-compound scoring time by several orders of magnitude relative to direct AutoDock Vina docking, with this advantage growing further as library size scales toward the billions of compounds available in modern make-on-demand libraries (Section 2.8, Table 4).

## Figures and Tables

**Table 1:** Effect of neural network width and depth on SurroDock performance for 1GWR and 3ERT.

Target	Database	Model	Width	Depth	RMSE ± SD	R^2^ ± SD
1GWR	NCI Open	M5	1024	2	1.1153 ± 0.1201	0.8417 ± 0.0034
			2048	2	1.1196 ± 0.1351	0.8405 ± 0.0099
			4096	2	1.1139 ± 0.1271	0.8412 ± 0.0079
			1024	3	1.1057 ± 0.1230	0.8430 ± 0.0095
			2048	3	1.1075 ± 0.1355	0.8423 ± 0.0174
			4096	3	1.1267 ± 0.0899	0.8368 ± 0.0093
			2048	4	1.1272 ± 0.0981	0.8355 ± 0.0120
			4096	4	1.1154 ± 0.1365	0.8412 ± 0.0102
3ERT	NCI Open	M5	1024	2	0.4469 ± 0.0355	0.9281 ± 0.0042
			2048	2	0.4497 ± 0.0523	0.9250 ± 0.0046
			4096	2	0.4566 ± 0.0220	0.9247 ± 0.0045
			1024	3	0.4473 ± 0.0223	0.9278 ± 0.0052
			2048	3	0.4468 ± 0.0470	0.9255 ± 0.0023
			4096	3	0.4672 ± 0.0337	0.9211 ± 0.0081
			2048	4	0.4626 ± 0.0338	0.9233 ± 0.0062
			4096	4	0.4754 ± 0.0492	0.9194 ± 0.0080

**Table 2:** Effect of Morgan fingerprint radius and bit vector length on SurroDock performance using the M5 feature set.

Target	Database	Model	Bits	FP Radius	RMSE ± SD	R^2^ ± SD
1GWR	NCI Open	M5	2048	2	1.1057 ± 0.1230	0.8430 ± 0.0095
			2048	3	1.1221 ± 0.1162	0.8393 ± 0.0062
			2048	4	1.1301 ± 0.0830	0.8345 ± 0.0068
			2048	5	1.1464 ± 0.1205	0.8308 ± 0.0128
			2048	6	1.1275 ± 0.0951	0.8362 ± 0.0093
			4096	2	1.1063 ± 0.1206	0.8427 ± 0.0074
			4096	3	1.1289 ± 0.1179	0.8372 ± 0.0097
3ERT	NCI Open	M5	2048	2	0.4473 ± 0.0223	0.9278 ± 0.0052
			2048	3	0.4498 ± 0.0328	0.9268 ± 0.0064
			2048	4	0.4541 ± 0.0419	0.9258 ± 0.0056
			2048	5	0.4550 ± 0.0313	0.9256 ± 0.0035
			2048	6	0.4578 ± 0.0257	0.9247 ± 0.0029
			4096	2	0.4493 ± 0.0422	0.9280 ± 0.0060
			4096	3	0.4480 ± 0.0290	0.9271 ± 0.0060

**Table 3:** Performance comparison across data-composition strategies under training, cross-validation, and held-out validation settings for the 1GWR and 3ERT targets.

Target	Data Set	Type	*n* (k)	*R* ^2^	RMSE	MAE	Pearson *r*	CI	Recall@1%
1GWR	NCI Open-only	TR	232	0.980	0.393	0.183	0.990	0.956	0.807
		CV	232	0.843	1.089	0.575	0.919	0.891	0.505
		HOV	102	0.753	1.391	0.855	0.869	0.846	0.389
		HOV-H	19	−2.834	0.972	0.630	0.338	0.657	0.337
	PubChem-only	TR	98	0.981	0.380	0.197	0.990	0.952	0.742
		CV	94	0.835	1.098	0.691	0.914	0.866	0.414
		HOV	236	0.495	1.977	0.909	0.758	0.841	0.410
		HOV-H	37	−0.131	1.426	0.723	0.229	0.668	0.208
	Mix-200k (1:1)	TR	200	0.977	0.404	0.196	0.989	0.952	0.805
		CV	194	0.830	1.083	0.623	0.912	0.882	0.442
		HOV	129	0.818	1.238	0.657	0.905	0.886	0.481
		HOV-H	25	−1.799	0.980	0.684	0.406	0.705	0.285
	Mix-150k (1:0.5)	TR	150	0.940	0.665	0.404	0.970	0.917	0.531
		CV	145	0.825	1.136	0.661	0.902	0.880	0.411
		HOV	179	0.776	1.304	0.691	0.894	0.875	0.440
		HOV-H	28	−2.333	1.049	0.607	0.527	0.694	0.218
	Mix-250k (1:1.5)	TR	250	0.976	0.429	0.228	0.988	0.948	0.734
		CV	244	0.844	1.099	0.618	0.919	0.886	0.503
		HOV	179	0.819	1.106	0.558	0.905	0.892	0.458
		HOV-H	17	−1.302	0.875	0.510	0.417	0.714	0.306
	Mix-300k (1:2)	TR	300	0.975	0.436	0.238	0.988	0.946	0.750
		CV	293	0.834	1.105	0.620	0.910	0.884	0.478
		HOV	29	0.832	1.103	0.554	0.912	0.900	0.479
		HOV-H	7	−0.728	0.811	0.476	0.498	0.741	0.338
	Unified-150k	TR	150	0.981	0.383	0.196	0.990	0.954	0.821
		CV	148	0.819	1.169	0.655	0.906	0.879	0.481
		HOV	179	0.873	0.978	0.524	0.934	0.904	0.552
		HOV-H	29	−1.086	0.789	0.484	0.487	0.728	0.444
	Unified-200k	TR	200	0.980	0.390	0.206	0.990	0.953	0.782
		CV	197	0.826	1.147	0.640	0.910	0.881	0.465
		HOV	129	0.881	0.943	0.505	0.935	0.906	0.573
		HOV-H	42	−0.767	0.726	0.452	0.613	0.729	0.469
	Unified-225k	TR	225	0.978	0.405	0.223	0.989	0.949	0.764
		CV	221	0.826	1.147	0.641	0.910	0.881	0.484
		HOV	104	0.886	0.923	0.496	0.942	0.908	0.582
		HOV-H	17	−0.544	0.683	0.422	0.566	0.737	0.476
	Unified-250k	TR	250	0.983	0.361	0.187	0.991	0.956	0.812
		CV	245	0.836	1.113	0.614	0.915	0.885	0.498
		HOV	79	0.892	0.905	0.482	0.944	0.910	0.598
		HOV-H	13	−0.560	0.688	0.431	0.557	0.739	0.484
3ERT	NCI Open-only	TR	236	0.947	0.382	0.248	0.973	0.928	0.622
		CV	236	0.929	0.444	0.286	0.964	0.917	0.381
		HOV	102	0.837	0.456	0.337	0.915	0.867	0.242
		HOV-H	19	0.386	0.513	0.384	0.690	0.752	0.560
	PubChem-only	TR	98	0.978	0.163	0.106	0.989	0.949	0.775
		CV	94	0.872	0.391	0.296	0.934	0.878	0.829
		HOV	241	0.497	1.185	0.511	0.723	0.869	0.342
		HOV-H	67	0.326	0.583	0.426	0.681	0.750	0.407
	Mix-150k (1:0.5)	TR	150	0.958	0.280	0.160	0.981	0.947	0.811
		CV	145	0.898	0.435	0.300	0.948	0.901	0.540
		HOV	183	0.901	0.524	0.330	0.952	0.910	0.523
		HOV-H	50	0.290	0.580	0.406	0.683	0.756	0.435
	Mix-200k (1:1.0)	TR	200	0.969	0.260	0.151	0.985	0.949	0.529
		CV	194	0.915	0.428	0.291	0.957	0.901	0.805
		HOV	134	0.911	0.502	0.309	0.954	0.911	0.517
		HOV-H	43	0.359	0.562	0.396	0.711	0.764	0.456
	Mix-250k (1:1.5)	TR	250	0.972	0.255	0.148	0.986	0.949	0.782
		CV	243	0.919	0.435	0.290	0.959	0.908	0.551
		HOV	84	0.919	0.461	0.291	0.919	0.916	0.554
		HOV-H	29	0.460	0.521	0.376	0.736	0.773	0.443
	Mix-300k (1:2)	TR	300	0.977	0.236	0.124	0.989	0.958	0.836
		CV	293	0.926	0.421	0.283	0.962	0.914	0.564
		HOV	34	0.903	0.498	0.294	0.951	0.915	0.559
		HOV-H	13	0.475	0.520	0.366	0.749	0.777	0.545
	Unified-150k	TR	150	0.976	0.115	0.115	0.988	0.959	0.857
		CV	148	0.912	0.310	0.310	0.955	0.905	0.521
		HOV	184	0.930	0.411	0.267	0.965	0.956	0.589
		HOV-H	58	0.475	0.475	0.342	0.774	0.748	0.546
	Unified-200k	TR	200	0.978	0.230	0.148	0.989	0.950	0.812
		CV	197	0.921	0.436	0.294	0.960	0.909	0.743
		HOV	134	0.932	0.408	0.264	0.965	0.920	0.606
		HOV-H	42	0.504	0.463	0.330	0.776	0.783	0.573
	Unified-225k	TR	225	0.974	0.251	0.156	0.987	0.949	0.812
		CV	221	0.921	0.438	0.295	0.960	0.908	0.777
		HOV	109	0.932	0.405	0.261	0.966	0.921	0.618
		HOV-H	34	0.500	0.465	0.331	0.783	0.787	0.595
	Unified-250k	TR	250	0.970	0.269	0.143	0.985	0.961	0.860
		CV	245	0.924	0.429	0.288	0.961	0.910	0.819
		HOV	84	0.934	0.401	0.252	0.967	0.923	0.623
		HOV-H	26	0.551	0.441	0.312	0.796	0.791	0.596

TR: training-set performance. CV: 5-fold cross-validation performance. HOV: held-out validation performance on compounds not used for model training. HOV-H: favorable-docking-score subset of HOV with reference Vina scores satisfying ΔG_Vina_ < −8.0 kcal/mol. *n* (k): number of compounds in thousands. Recall@1%: fraction of reference Vina top-1% compounds recovered in the SurroDock-predicted top-1% set. CI: concordance index, where 0.5 indicates random ordering, and 1.0 indicates perfect concordance.

**Table 4:** Computational cost comparison of direct AutoDock Vina docking and SurroDock screening.

Method	Target	No. of Compounds	Training Time	Docking/Prediction Time	Time per Compound	Hardware
Autodock Vina	1GWR	200,000	-	1007 CPU-core-h	18.1 s	CPU
	3ERT	200,000	-	3098 CPU-core-h	55.8 s	CPU
SurroDock	1GWR	200,000	24.2 min	10.0 s	0.050 ms	GPU
	3ERT	200,000	16.4 min	10.1 s	0.051 ms	GPU

AutoDock Vina costs were normalized to 200,000 compounds using NCI-derived mean runtimes: 18.13 s/compound for 1GWR and 55.77 s/compound for 3ERT. SurroDock values correspond to the representative M5 model. Calculations were performed on a workstation with an Intel Core i9-9900K CPU, 32 GB RAM, and an NVIDIA GeForce RTX 2080 Ti 11 GB GPU under Ubuntu 24.04.1 LTS.

## Data Availability

The raw data supporting the conclusions of this article will be made available by the author on request.

## Supplementary Materials

The following supporting information can be downloaded at: https://www.mdpi.com/article/10.3390/ijms27156663/s1.
