# Supplementary material for: SurroDock: A Deep Learning Surrogate for Accelerated Pre-Docking Ligand Prioritization in Structure-Based Virtual Screening
Source: Int J Mol Sci. 2026 Jul 26;27(15):6663. doi: 10.3390/ijms27156663 (PMC13466497; doi:10.3390/ijms27156663)
Supplement: Supplementary file 1 [file ijms-27-06663-s001.zip › ijms-4413663-supplementary.pdf]

Supplementary

SurroDock: A Deep Learning Surrogate for Accelerated Pre-Docking Ligand Prioritization in Structure-Based Virtual Screening

Jongkeun Choi

Department of Chemical & Biological Engineering, Chungwoon University, Incheon 22100, Republic of Korea; jkchoi@chungwoon.ac.kr

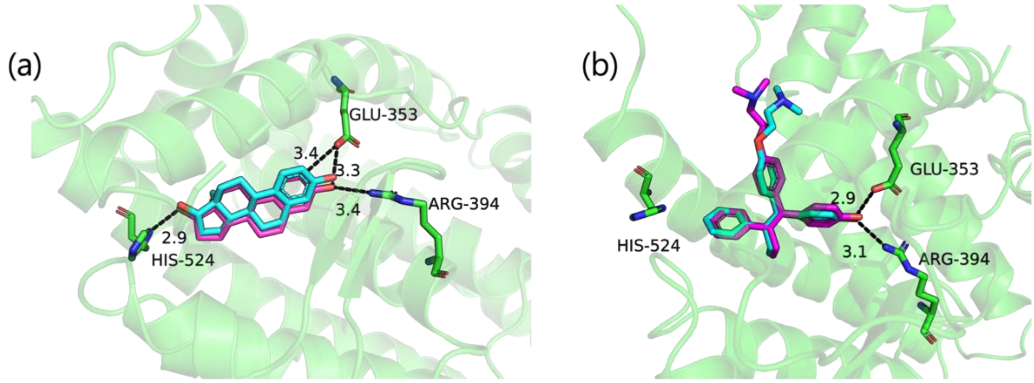

Figure S1. Comparative binding mode analysis of agonist and antagonist in the ERα active site. Superposition of the docked poses (cyan) and co-crystallized structures (magenta) for (a) Estradiol (agonist) and (b) 4-Hydroxytamoxifen (antagonist, PDB: 3ERT). Hydrogen bonds are indicated by black dashed lines. The calculated RMSD values are 0.7212 Å and 1.250 Å, respectively, demonstrating a high degree of structural similarity. Hydrogen atoms are omitted for visual clarity; the tertiary amine within the dimethylaminoethoxy side chain of 4-hydroxytamoxifen was docked in its protonated form, consistent with its expected ionization state at physiological pH.

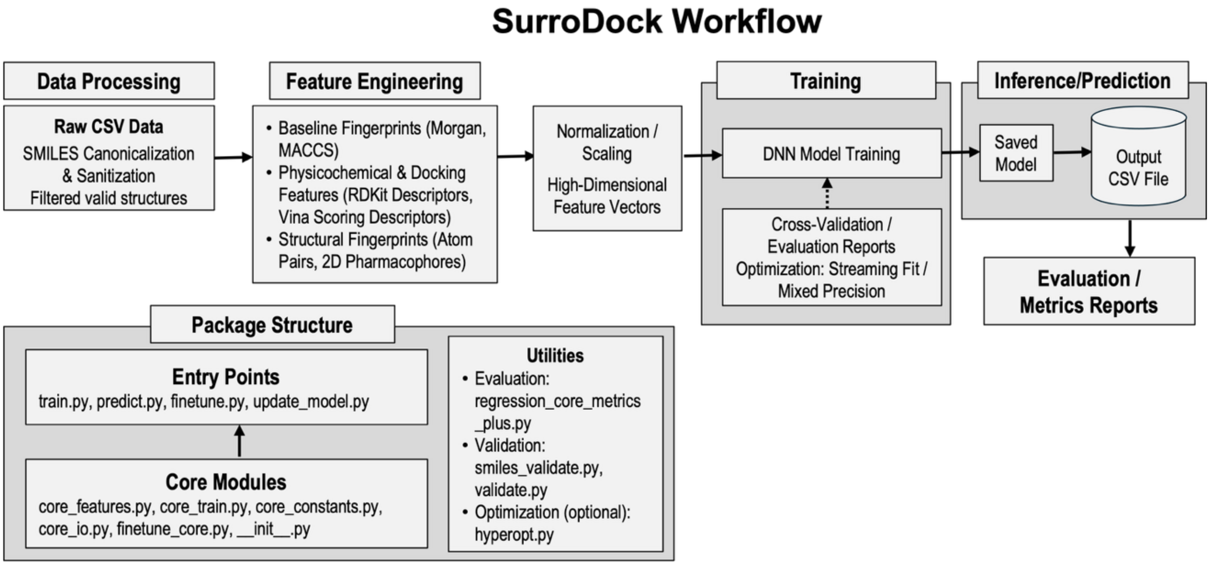

Figure S2. SurroDock workflow. The pipeline proceeds from raw CSV ingestion and SMILES canonicalization/sanitization to feature engineering, descriptor scaling, high-dimensional feature-vector assembly, model training, cross-validation/evaluation, and inference. The trained model is used to generate predicted AutoDock Vina docking scores, which are exported together with performance metrics and prediction reports.

```
surrodock/
│
├── Entry Points (User Interface)
│   ├── train.py            # Main training pipeline
│   ├── predict.py          # Inference engine
│   ├── finetune.py         # Transfer learning
│   └── cli.py              # Argument parser
│
├── Core Modules (Backend Logic)
│   ├── core_features.py    # Feature engineering
│   ├── core_train.py       # Model architecture
│   ├── core_cv.py          # Cross-validation
│   └── core_io.py          # Data I/O & Normalization
│
└── Utilities
    ├── config_constants.py
    └── regression_core_metrics_plus.py
```

Figure S3. SurroDock software architecture and module dependencies. The package separates user-facing command-line entry points from backend modules for data I/O, feature generation, model construction, cross-validation, inference, metric calculation, validation, and optional hyperparameter optimization.

Table S1. Key modules and functionality of the SurroDock software package.

Module Category	File Name	Key Functionality
**Entry Points**	train.py	Executes the end-to-end training pipeline.
	predict.py	Performs inference on new datasets.
	finetune.py	Fine-tunes pre-trained models for specific targets.
	cli.py	Handles command-line arguments and environment setup.
**Core Logic**	core_features.py	Generates fingerprints and assembles feature vectors.
	core_train.py	Defines DNN architecture and "Streaming Fit" loop.
	core_cv.py	Manages K-Fold Cross-Validation splitting.
	core_io.py	Handles data loading, normalization, and model saving.
**Utilities**	config_constants.py	Stores global presets and path configurations.
	regression_core_metrics_ plus.py	Custom regression metrics (R^2, RMSE, etc.).

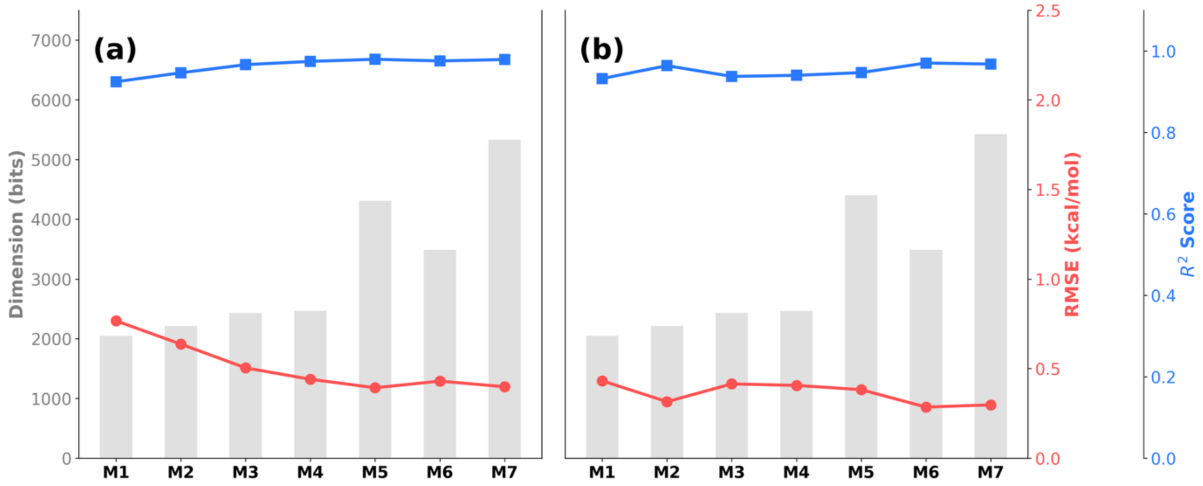

Figure S4. Performance evaluation of cumulative feature combinations on the 1GWR and 3ERT datasets using whole-dataset fitting evaluation. (a) 1GWR and (b) 3ERT results, in which each model was trained and evaluated on the entire dataset to assess maximum fitting capacity rather than generalization performance. Gray bars represent feature dimensionality (bits, left axis), while red circles and blue squares indicate RMSE (kcal/mol) and R^2 values, respectively. The cumulative feature sets are defined as follows: M1, Morgan fingerprints; M2, M1 + MACCS keys; M3, M2 + RDKit physicochemical descriptors; M4, M3 + Vina-inspired docking-related descriptors; M5, M4 + atom-pair fingerprints; M6, M4 + 2D pharmacophore fingerprints; and M7, all feature classes combined.

Table S2. Effect of Morgan fingerprint radius and bit vector length on SurroDock performance. Experiments were performed using the M3 feature set to isolate the contribution of Morgan fingerprint parameters from other feature classes. The selected configuration (2048 bits, radius 2) was subsequently applied to all feature sets including M5.

Target	Database	Model	bits	FP radius	RMSE ± SD	R^2 ± SD
1GWR	NCI Open	M3	2048	2	1.1853 ± 0.1215	0.8191 ± 0.0101
			2048	3	1.1822 ± 0.1982	0.8238 ± 0.0188
			2048	4	1.2105 ± 0.1081	0.8103 ± 0.0105
			2048	5	1.2201 ± 0.1303	0.8077 ± 0.0085
			2048	6	1.2117 ± 0.1282	0.8103 ± 0.0085
			4096	2	1.1727 ± 0.1167	0.8236 ± 0.0045
			4096	3	1.1861 ± 0.1289	0.8192 ± 0.0097
3ERT	NCI Open	M3	2048	2	0.4539 ± 0.0260	0.9254 ± 0.0045
			2048	3	0.4643 ± 0.0422	0.9228 ± 0.0049
			2048	4	0.4679 ± 0.0327	0.9213 ± 0.0038
			2048	5	0.4683 ± 0.0325	0.9208 ± 0.0062
			2048	6	0.4729 ± 0.0402	0.9187 ± 0.0109
			4096	2	0.4534 ± 0.0352	0.9260 ± 0.0061
			4096	3	0.4665 ± 0.0346	0.9232 ± 0.0033

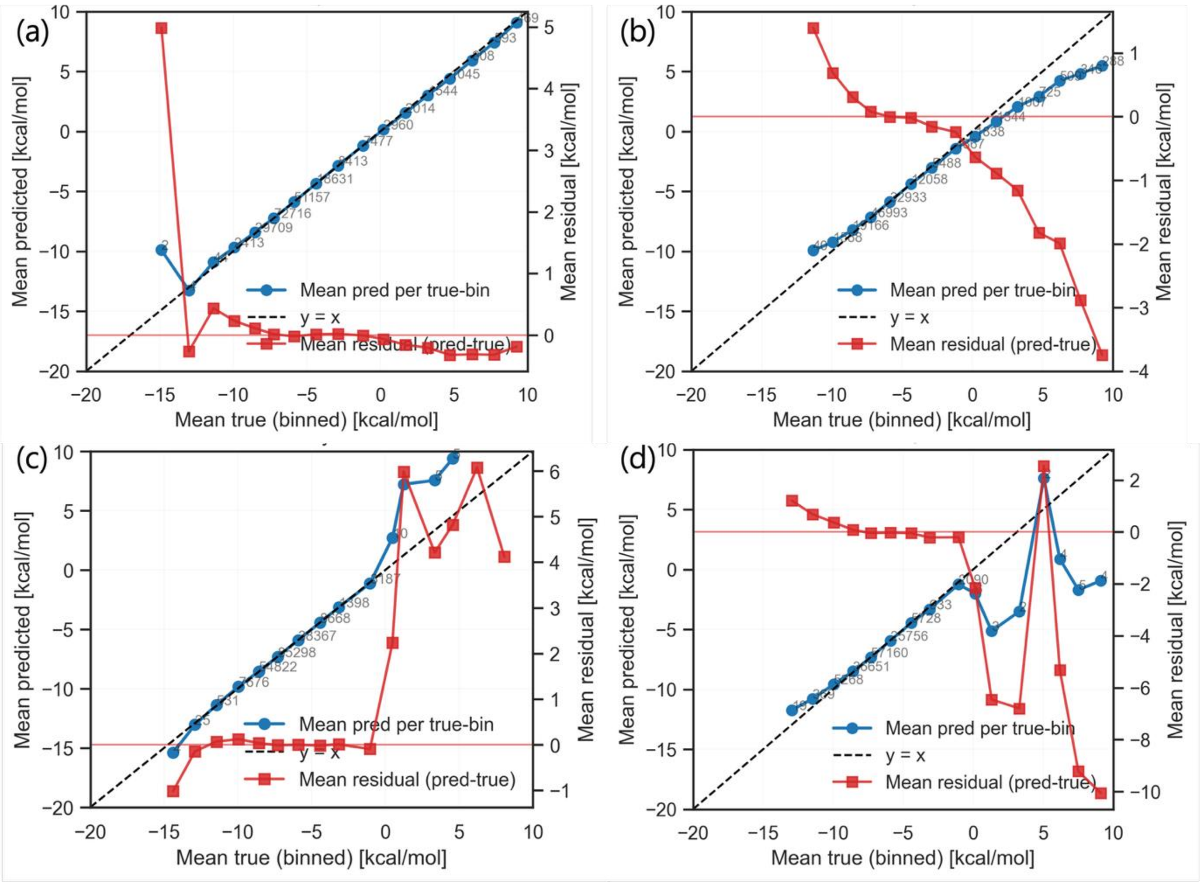

Figure S5. Calibration analysis across reference Vina-score ranges. Binned calibration plots compare the mean SurroDock-predicted score, blue circles and left y-axis, with the mean reference Vina score on the x-axis. Red squares and the right y-axis show the mean residual ($y_{pred} - y_{true}$). The black dashed line indicates ideal calibration, y =x, and the red horizontal line indicates zero mean residual. (a,b) Results for 1GWR on (a) the training set and (b) the held-out validation, HOV, set. (c,d) Results for 3ERT on (c) the training set and (d) the HOV set. The model is well calibrated in the central score range, where most compounds are distributed, but shows regression-to-the-mean behavior in sparsely populated extreme-score bins. These deviations indicate that predictions in highly favorable or unfavorable Vina-score regions should be interpreted with caution.

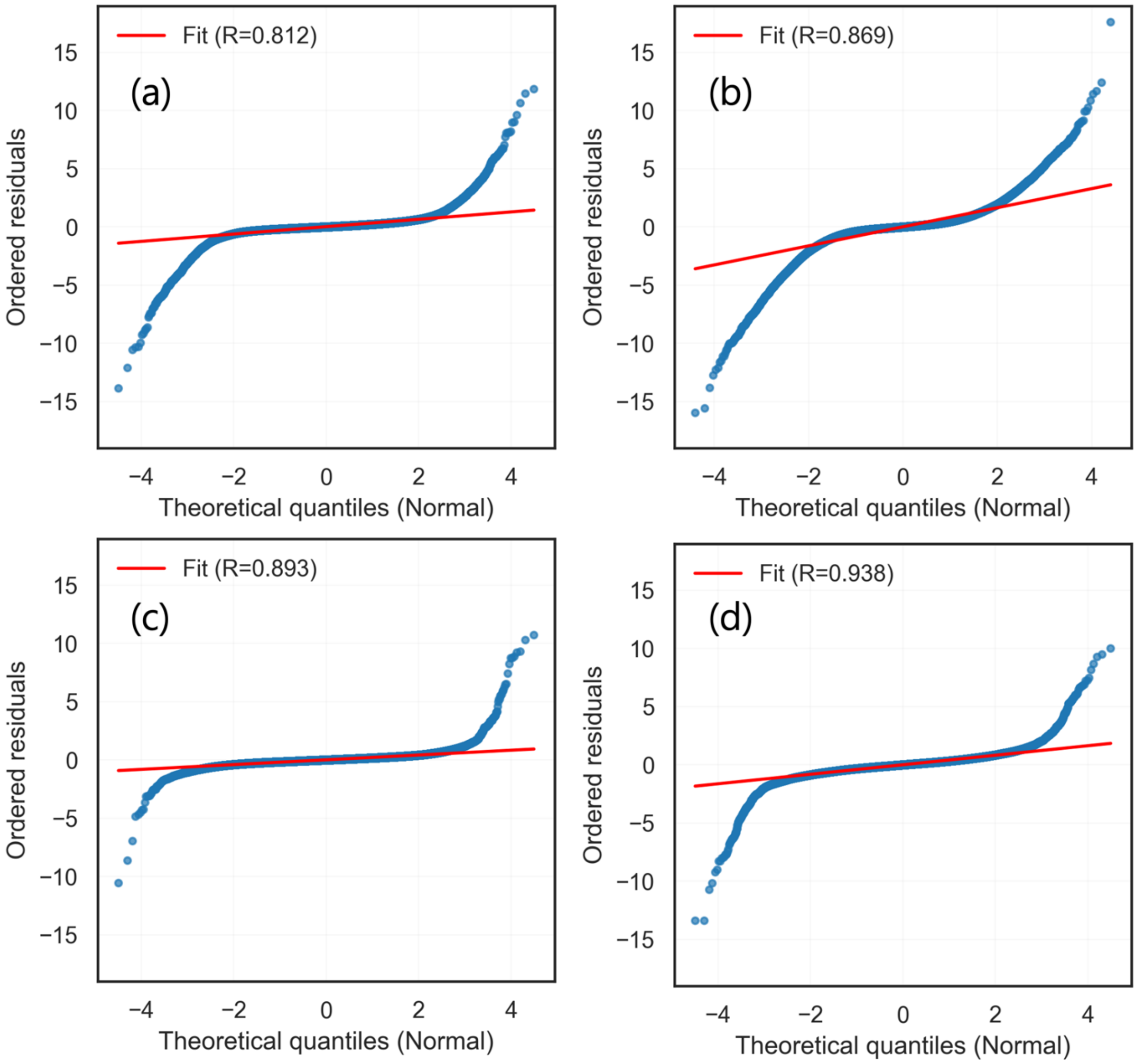

Figure S6. Quantile–quantile analysis of prediction residuals. Q–Q plots compare ordered prediction residuals ($y_{pred} - y_{true}$) with theoretical quantiles of a standard normal distribution. The red line indicates the least-squares reference fit, and the reported R value denotes the Pearson correlation between the ordered residuals and theoretical normal quantiles. (a,b) Results for 1GWR on (a) the training set and (b) the held-out validation, HOV, set. (c,d) Results for 3ERT on (c) the training set and (d) the HOV set. The S-shaped deviations from the reference line indicate heavy-tailed residual distributions, meaning that extreme errors occur more frequently than expected under a Gaussian error model. This behavior is consistent with the presence of rare large-error outliers and indicates that the MSE-trained model underweights extreme residuals. For compounds in the favorable-score tail, the heavy-tailed residual distribution reinforces the recommendation for confirmatory docking prior to experimental follow-up.

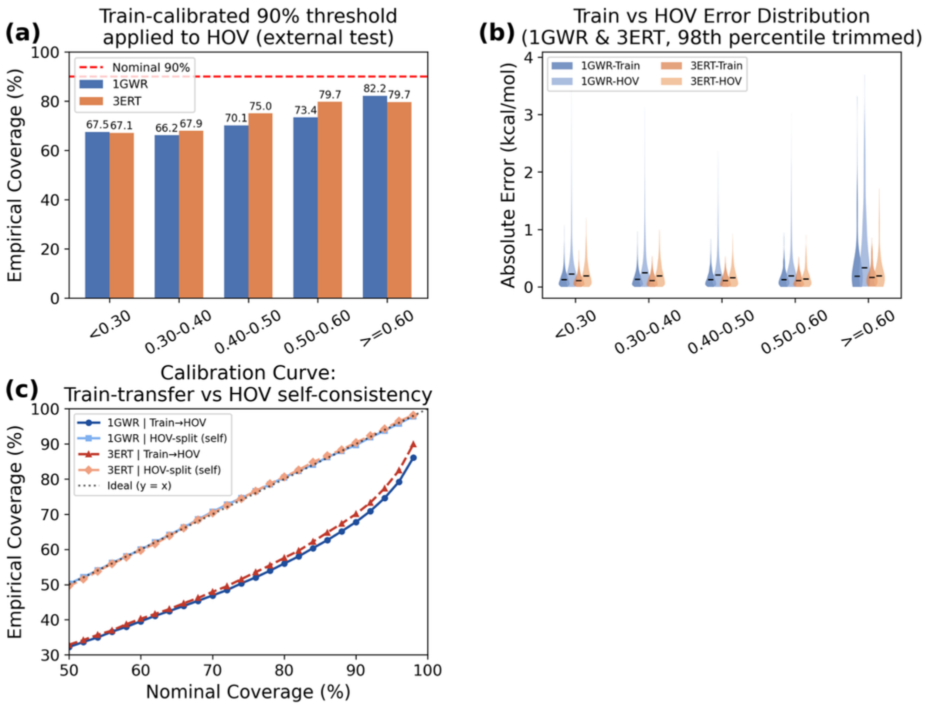

Figure S7. Similarity-stratified conformal prediction analysis of SurroDock scoring error, testing whether prediction reliability calibrated on the training set carries over to unseen (HOV) compounds, for two representative targets (1GWR and 3ERT). Absolute scoring errors (|predicted - true docking score|) were binned by nearest-neighbor Tanimoto similarity (Tc) to the training set into five strata (<0.30, 0.30-0.40, 0.40-0.50, 0.50-0.60, >=0.60). We adopted a Mondrian, or group-conditional, conformal prediction scheme, calibrating separately within each similarity bin rather than pooling all compounds together.

(a) Empirical coverage on HOV when a 90% error threshold is fit on the training set within each similarity bin and then applied as-is - no refitting - to the corresponding HOV bin (bar plot, values labeled above each bar; red dashed line marks the nominal 90% target). If coverage falls short of 90%, the Train-derived threshold was too optimistic for that similarity range once applied externally; coverage at or above 90% means the threshold still holds up on unseen chemistry.

(b) Absolute-error distributions for each similarity bin, shown as violin plots comparing Train (dark) against HOV (light) for both targets. The top 2% of errors were trimmed purely for visual clarity and do not affect any reported statistics. These are the raw distributions behind the thresholds and coverage numbers in (a), and make it easy to see where the median error or tail width actually shifts between Train and HOV.

(c) Calibration curves for two different transfer setups, pooling across all similarity levels. "Train->HOV" (circle/triangle markers) calibrates on the training set and checks coverage on the full HOV set; "HOV-split (self-consistency)" (square/diamond markers) instead splits HOV itself in half, calibrating on one half and te

sting on the other. The latter has no train-to-test distribution shift by construction, so it works as a best-case benchmark for how well conformal calibration can perform even under ideal conditions. Because the Train->HOV curve stays close to this HOV-split reference across most of the coverage range, the calibration method itself does not appear to be the problem - the gap that does show up, concentrated in the low-similarity bins from panel (a), more likely reflects a genuine shift in chemical space between training and held-out compounds. The dotted diagonal marks perfect calibration (nominal = empirical).

Taken together, the three panels suggest that SurroDock's bin-wise error estimates stay reasonably trustworthy - sometimes a bit conservative, occasionally a bit optimistic - even for compounds structurally far from the training set, which is the basis for using similarity-aware error bounds as a practical reliability check during prospective screening (see main text, Section 2.9).

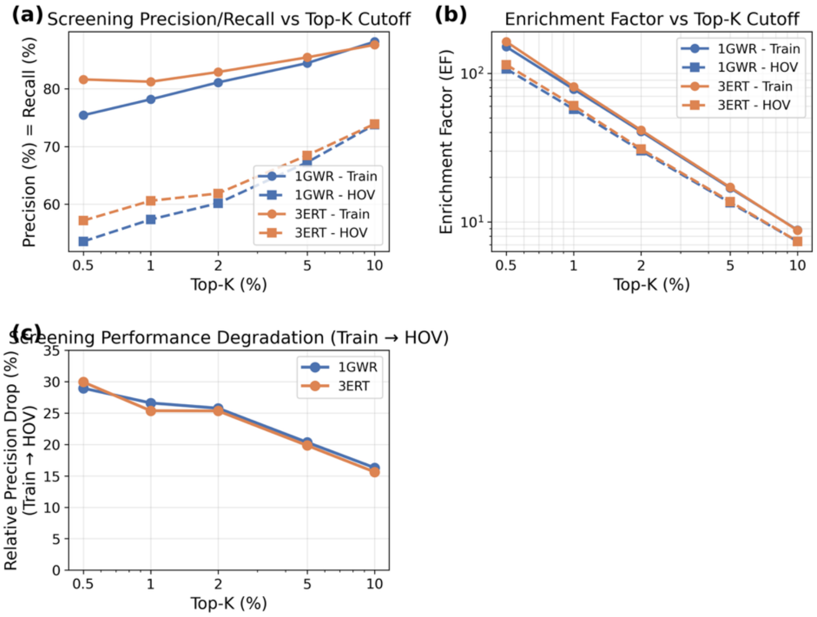

Figure S8. Top-K virtual screening performance of SurroDock on the training set versus the hold-out validation (HOV) set, evaluated on two representative targets (1GWR and 3ERT). For each target and split, compounds were ranked by SurroDock-predicted docking score, and the top-K% of that ranking was compared against the top-K% obtained from true (docked) scores - this tells us how faithfully the surrogate reproduces the actual top-ranked hit list at selection thresholds that matter in practice (K = 0.5, 1, 2, 5, and 10%).

(a) Screening precision and recall as a function of the Top-K cutoff. Since both the predicted and true rankings select the same fixed number of compounds at each K, precision and recall come out numerically identical at every cutoff. Solid lines with circular markers show training-set performance; dashed lines with square markers show HOV performance. Both targets follow the expected pattern - precision/recall rises with larger K - and the gap between Train and HOV narrows as K grows.

(b) Enrichment factor (EF), i.e., the true-positive rate within the Top-K selection relative to what random selection would give, plotted against Top-K cutoff on a log-log scale. EF peaks at the smallest K (0.5%), which makes sense: even under the strictest selection, the truly top-ranked compounds are being enriched strongly, and EF falls off monotonically as K grows. HOV tracks Train closely across nearly the whole range of K, so enrichment power appears to carry over well to unseen compounds.

(c) Relative precision drop going from Train to HOV, calculated as (Precision_Train - Precision_HOV) / Precision_Train x 100 (%) - essentially compressing the generalization gap from panel (a) into one number. The drop is largest at the tightest cutoff (roughly 29-30% at K = 0.5%) and shrinks steadily as K increases, down to about 16% at K = 10% for both targets. In other words, the surrogate has some trouble nailing the exact smallest set of top hits on unseen data, but widening the selection margin - somewhere in the 5- to 10-fold range above the intended screening fraction - closes most of that gap. This is the basis for recommending such a margin as a practical rule of thumb for prospective screening (see main text, Section 2.9).

Table S3. Mondrian split-conformal 90% prediction-interval (PI) half-widths calibrated on the docked training subset, and the resulting empirical coverage when the Train-derived thresholds are applied to the hold-out validation (HOV) set, stratified by kNN Tanimoto similarity to the training subset (ksim), for targets 1GWR and 3ERT.

(A) Train-calibrated 90% conformal PI half-width

kNN similarity (k_{sim}) bin	1GWR n_{train}	1GWR PI90 (±kcal/mol)	3ERT n_{train}	3ERT PI90 (±kcal/mol)
< 0.30	5,471	0.409	5,553	0.307
0.30 – 0.40	16,012	0.425	16,057	0.300
0.40 – 0.50	7,660	0.397	7,517	0.299
0.50 – 0.60	774	0.444	748	0.306
≥ 0.60	83	1.490	125	0.560

(B) Empirical coverage on HOV using Train-derived thresholds (target: 90%)

kNN similarity (k_{sim}) bin	1GWR n_{HOV}	1GWR Coverage	3ERT n_{HOV}	3ERT Coverage
< 0.30	5,349	67.5%	5,484	67.1%
0.30 – 0.40	15,954	66.2%	15,981	67.9%
0.40 – 0.50	7,784	70.1%	7,632	75.2%
0.50 – 0.60	812	73.4%	775	79.7%
≥ 0.60	101	82.2%	128	82.8%

Note. PI half-widths (Panel A) were computed via split-conformal calibration (nonconformity score = absolute residual) on the docked training subset within each similarity bin, using the $\lceil(n+1)(1-\alpha)\rceil/n$-th empirical quantile of |predicted − true| ($\alpha = 0.10$), capped at the 100th percentile when the bin size was small. Panel B reports the fraction of HOV observations in each bin whose absolute residual falls within the Train-derived threshold from Panel A; values substantially below the 90% target indicate distribution shift between the Train and HOV splits rather than a methodological failure of the conformal procedure itself. Bins with high similarity (≥ 0.60) show coverage closest to nominal for both targets, consistent with the interpretation that the Train/HOV shift is most pronounced in the low-to-mid similarity regime (k_sim < 0.50). See Supplementary Figure S8 for the corresponding calibration curves and Supplementary Figure S7 for the Top-K screening margin analysis that defines the primary actionable selection criterion for prospective screening.
